# Genomic analysis identified a potential novel molecular mechanism for high-altitude adaptation in sheep at the Himalayas

**DOI:** 10.1038/srep29963

**Published:** 2016-07-22

**Authors:** Neena Amatya Gorkhali, Kunzhe Dong, Min Yang, Shen Song, Adiljian Kader, Bhola Shankar Shrestha, Xiaohong He, Qianjun Zhao, Yabin Pu, Xiangchen Li, James Kijas, Weijun Guan, Jianlin Han, Lin Jiang, Yuehui Ma

**Affiliations:** 1 Institute of Animal Science, Chinese Academy of Agricultural Sciences (CAAS), No. 2 Yuanmingyuan West Road, Beijing 100193, China; 2 Animal Breeding Division, National Animal Science Institute, Nepal Agriculture Research Council (NARC), Kathmandu, Nepal; 3 CAAS-ILRI Joint Laboratory on Livestock and Forage Genetic Resources, Institute of Animal Science, Chinese Academy of Agricultural Sciences (CAAS), Beijing 100193, China; 4 Department of Animal Genetics and Breeding, China Agricultural University, Beijing 100094, P. R. China; 5 CSIRO Livestock Industries, St Lucia, Brisbane, Qld, Australia; 6 International Livestock Research Institute (ILRI), P.O. Box 30709, Nairobi 00100, Kenya

## Abstract

Sheep has successfully adapted to the extreme high-altitude Himalayan region. To identify genes underlying such adaptation, we genotyped genome-wide single nucleotide polymorphisms (SNPs) of four major sheep breeds living at different altitudes in Nepal and downloaded SNP array data from additional Asian and Middle East breeds. Using a *d*_*i*_ value-based genomic comparison between four high-altitude and eight lowland Asian breeds, we discovered the most differentiated variants at the locus of *FGF-7* (*Keratinocyte growth factor-7*), which was previously reported as a good protective candidate for pulmonary injuries. We further found a SNP upstream of *FGF-7* that appears to contribute to the divergence signature. First, the SNP occurred at an extremely conserved site. Second, the SNP showed an increasing allele frequency with the elevated altitude in Nepalese sheep. Third, the electrophoretic mobility shift assays (EMSA) analysis using human lung cancer cells revealed the allele-specific DNA-protein interactions. We thus hypothesized that *FGF-7* gene potentially enhances lung function by regulating its expression level in high-altitude sheep through altering its binding of specific transcription factors. Especially, *FGF-7* gene was not implicated in previous studies of other high-altitude species, suggesting a potential novel adaptive mechanism to high altitude in sheep at the Himalayas.

Sheep (*Ovis aries*) is one of the first domesticated livestock species whose ancestors mainly roosted in the Fertile Crescent regions of Southwest Asia around 10,000 years ago^1^,^2^. They then spread west throughout Europe, south into North Africa and east into Asia alongside their human domesticators^3^. Abundant sheep genetic resources were thus formed in different ecological niches along their spreading routes. Among them, sheep living in the Himalayas, specifically in Nepal, Tibet and Ladakh, provides an outstanding animal model to study the genetic and molecular mechanism for the high-altitude adaptation because of the acute altitudinal transition in these regions.

The Himalayan mountain range extends up to Tibetan plateau on the north and is bordered on the south by the Indo-Gangetic Plain, with Nepal lying between them (Fig. 1). Nepal thus has a spectacular altitudinal range from less than 80 m above sea level (masl) in the south to 8,848 masl on the northern border. There are four major breeds including Bhyanglung, Baruwal, Kage and Lampuchhre, contributing more than 90% of the total sheep population (Fig. 1). The habitat of Bhyanglung sheep is located at the north to Himalayas, which further stretch northward to the Tibetan plateau, covering most of the Tibetan Autonomous Region and Qinghai Province (habitat for Tibetan sheep) in western China, as well as part of Ladakh (habitat for Changthangi sheep, another high-altitude sheep breed) (Fig. 1). Baruwal sheep is the principal breed in Nepal, representing 63% of the total sheep population and is well-adapted to quite a range of altitudes from 2000 to 4000 masl^4^. Kage and Lampuchhre sheep are two lowland breeds, distributed in the areas below 1500 masl. An earlier phylogenetic studies using blood proteins showed that Bhyanglung, as well as Tibetan and Changthangi sheep were derived from the Tibetan sheep group, and Baruwal belongs to Himalayan group, while Kage and Lampuchhre are associated with Indian group^5^. However, no genome-wide analysis was performed to examine the intra- and/or inter-population genetic variability or to detect the selection signatures for the high-altitude adaptation among these four major Nepalese sheep.

The adaptation to high-altitude environment is believed to be a result of advantageous genetic mutations and selective pressure. For identifying the alleles that have been subject to selection, a variety of statistical methods have been developed, mainly based on (i) the high frequency of derived alleles and the consequences of hitchhiking within population, such as Fay and Wu’s *H* Test^6^; (ii) the length and structure of haplotypes by applying either EHH^7^, iHS^8^ or Rsb^9^; (iii) the genetic differentiation between populations, measured by F_ST_ or the related statistics^10^. Based on F_ST_, a statistic termed *di* was recently developed to detect selective events in dog genome^11^. *Di* is defined as a function of unbiased estimates of all pairwise F_ST_ between one breed and the remaining breeds within a population. It is particularly suited for detecting selection specific to a particular breed, or subset of breeds, and isolating the direction of change. Using these methods, candidate genes that contributed to the high-altitude adaptation in human^12^,^13^,^14^,^15^,^16^,^17^, yak^18^, Tibetan antelope^19^, grey wolf^20^, dog^21^,^22^,^23^, pig^24^,^25^,^26^, chicken^27^ and goat^28^ have been identified. A number of responsible genes have been proposed by these reports and among them, the most prominent ones were *EPAS1* (endothelial PAS domain protein1; also known as HIF2A) and *EGLN1* (egl-9 family hypoxia inducible factor 1; also known as HIF prolylhydroxylase 2, PHD2). Both candidates are the key genes functioning at the upstream of the hypoxia inducible factor (HIF) pathway and the functional mutations of these two genes have been documented^29^,^30^. Generally, these studies showed that convergent evolution appears to have shaped the similar group of genes in the adaptive process of different species, such as the *EPAS1* gene shared by Tibetans^14^,^16^,^17^, Tibetan mastiff^21^,^22^,^23^, Tibetan grey wolf^20^ and Tibetan goat^28^. On the other hand, even for the same species, different geographic populations with divergent genetic background have unique adaptive mechanisms, examples including human (from Tibet, Andes and Ethiopia)^12^,^15^,^16^ and Tibetan pig (from Tibet, Gansu, Sichuan and Yunnan province in China)^25^. The genetic mechanism of high-altitude adaptation in sheep, one of the most commonly distributed livestock, however, remains perplexing.

To delve into these issues, we genotyped the four major Nepalese sheep breeds comprising of two high-altitude breeds (Bhyanglung and Baruwal), and two lowland breeds (Kage and Lampuchhre) using Illumina *ovine* 50KSNP Beadchip. We then downloaded the publicly available SNP beadchip data from the other two Tibetan-lineage sheep (Tibetan and Changthangi sheep) as well as 15 other breeds from Asian and Middle East. After merging with our data, we conducted a phylogenetic analysis and a genomic scan for signatures of directional selection in high-altitude sheep. Re-sequencing data of the candidate locus was analyzed to map the major variant and Nepalese sheep individuals were further screened for the variant.

## Results

We genotyped 59,450 SNPs using Illumina *Ovine* SNP50 beadchip array in a panel of 96 Nepalese sheep including two high-altitude breeds (Bhyanglung and Baruwal) and two low-land breeds (Lampuchhre and Kage), with each breed containing 24 individuals. To better understand the evolution of the sheep breeds at the Himalayan region in the context of their geographic neighbors, the SNP data of 454 sheep individuals from eight Asian and nine Middle East breeds were merged with our data, producing a common data set of 47,415 genotyped SNPs in 550 individuals (Table 1). After applying a series of quality control filters, a total of 45,184 autosomal SNPs were used in the subsequent analysis.

### Phylogenetic analyses

To examine the phylogenetic relationships among the sheep breeds, we first performed principle component analysis (PCA) based on the pruned genotype data of 36,711 SNPs from 21 Asian sheep breeds (n = 550) including nine Middle East, six Asian, one Tibetan, one Changthangi and four Nepalese sheep populations (Table 1). The first axis of the PCA (PC1) provided a good distinction between Middle East sheep and Asian sheep breeds (Fig. 2A). The second axis PC2 distinguished all the high-altitude breeds, including Bhyanglung, Tibetan, Changthangi and Baruwal, from the rest of the lowland sheep (Fig. 2A). Among the four high-altitude breeds, the Nepalese Bhyanglung perfectly clustered together with Tibetan and Changthangi to form the Tibetan group, yet was clearly separated by PC1 with the other Nepalese high-altitude breed (Baruwal) belonging to the Himalayan group, indicating a close but different genetic background between the two Nepalese high-altitude breeds. While the two Nepalese breeds from lowland, especially Lampuchhre, showed a relatively close relation to Deccani sheep from India. In addition, seven, five, three, four and one individuals were outside of their expected population clusters of Kage, Changthangi, Garut, Deccani and Sumatra sheep respectively, and were excluded from subsequent analyses.

A neighbor-joining tree using the same 21 Asian breeds agreed with the PCA analysis on the following aspects, including the evolutionary divergence between populations from Asia to Middle East, the close but different background between two high-altitude sheep groups, as well as the close relationship of Lampuchhre with the Indian group (Fig. 2B). Furthermore, the phylogenetic tree showed that Kage and Baruwal were clustered into one group, indicating Kage was closer to the Himalayan group than other breeds. This observation did not coincide with the results of PCA analysis, probably due to the shared ancestral variation and historical gene flow between them, or the bias of the algorithmic strategy.

To better understand the population variation, we performed Linkage Disequilibrium (LD) decay analysis, which can be informative for population demography. When we combined all our breeds, LD declined most rapidly (Supplementary Fig. S1), consistent with those observed in domestic cattle^31^, horse^32^ and dog^33^. This reflects a lack of conserved LD phase and haplotypes across our 21 Asian sheep breeds. The Tibetan group had low levels of LD, suggesting an ancient origin for this group. Baruwal had very high LD values across the range of distances separating loci, which suggested that they were derived from a relatively small ancestral population (Supplementary Fig. S1). The observation indicated quite different breeding histories between the Tibetan and Himalayan sheep groups.

### Identifying targets of selection in high-altitude sheep

To determine the extent of population differentiation between high- and low-altitude sheep breeds, we calculated the unbiased F_ST_ value at genome-wide level. The F_ST_ values for comparisons between the four high-altitude breeds (Bhyanglung, Tibetan, Changthangi and Baruwal) and the eight low-land breeds were 0.0809-0.1347, 0.0914-0.1426, 0.0805-0.133 and 0.1479-0.1991, respectively (Supplementary Tables S1). For detecting loci showing evidence of selection in high-altitude sheep breeds, we used a population based approach, termed *di* that exploits a biological contrast which in this study defined breeds as either high- or low-altitude (Table 1). We estimated *d*_i_ four times, in each case comparing one high-altitude population to a diverse collection of eight lowland breeds selected to maximize genetic diversity based on our phylogenetic analysis (Fig. 2B). The lowland breeds used are listed in Table 1. We defined those within the top 0.5% of the empirical distribution of *d*_i_ values as the candidate loci under selection for adaptation to high altitude, resulting in a total of 219 candidate loci per breed (Fig. 3A and Supplementary Tables S2–S5). And then, all significant SNPs within 500 kb of each other were merged into single regions, which yielded a total of 28, 36, 36 and 36 highly differentiated genomic regions, encompassing 5.09, 7.37, 6.18 and 5.34 Mb of the sheep genome in Bhyanglung, Tibetan, Changthangi and Baruwal, respectively (Supplementary Tables S6–S9). However, it is difficult to determine the exact genomic regions that have been subject to selection in high-altitude sheep breeds, due to ascertainment bias and limited number of genotyped SNPs. Whole-genome sequencing from multiple individuals would be necessary for validating this.

A total of 73, 97, 85 and 79 significant SNPs were located within the merged regions in Bhyanglung, Tibetan, Changthangi and Baruwal, respectively. Comparison of the four lists revealed that a large number of loci (n = 23) were shared by the three Tibetan-group breeds, consistent with the fact that these breeds have similar genetic background (Supplementary Fig. S2). Although a majority of candidate SNPs identified in Baruwal revealed uniqueness within breed, three SNPs were observed to be shared by all the other three high-altitude sheep breeds (Supplementary Fig. S2), indicating strong signatures of positive selection in high-altitude sheep breeds. For the three candidate SNPs, only one region (of less than 300 kb) on chromosome 7, contained more than one adjacent SNP (OAR7_63692612.1 and OAR7_63745942.1) (Fig. 3A).

Additionally, the selection signal on chromosome 7 was the strongest across genome among three Tibetan group populations (Fig. 3A). In Bhyanglung, Tibetan and Changthangi sheep, the peak signal contained four contiguous SNPs (OAR7_63692612.1, OAR7_63745942.1, OAR7_63814443.1 and OAR7_63848145.1), which appeared to be a strong selective sweep spanning 150.2-kb region (57,764,872 to 57,915,106 bp). This region contained two genes including *keratinocyte growth factor 7* (*FGF-7*) and *galactokinase 2* (*GALK2*). The four candidate SNPs in the Tibetan group with the exception of the SNP OAR7_63692612.1 in one breed (at the 90^th^ ranking), were all among the top 10 ranking SNPs with the highest *d*_*i*_ values (Table 2). While the peak signal in Baruwal sheep contained two (OAR7_63692612.1 and OAR7_63745942.1 that were at 148^th^ and 30^th^ ranking, respectively) of the four candidate SNPs mentioned above and defined a shorter region spanning 51.6-kb (57,764,872 to 57,816,492 bp) that only contained the *FGF-7* gene. Figure 3B provided the detailed frequencies of the major allele in high-altitude sheep of the four SNPs for each tested population.

To test additional loci not located on the ovine SNP50 chip across the *FGF-7* gene region for evidence of selection, we retrieved the International Sheep Genome Consortium (ISGC) re-sequencing data (http://projects.ensembl.org/nextgen/) of the most evident gene, *FGF-7,* from 12 sheep individuals comprising of two Tibetan, two Changthangi and eight other Asian sheep individuals (Supplementary Table S10). This identified a total of 329 SNPs (MAF > 0.05) in this region and 23 among them showed the significant difference in allele frequencies (P value < 0.001, Fisher’s exact test in Fig. 4A) between the four high-altitude sheep and their geographic relationship relatives (Supplementary Table S11). Each of these SNPs with significantly skewed allele frequencies were either intergenic (n = 2) or intronic (n = 21) according to the ensemble gene model (Fig. 4A). This offers preliminary evidence showing that the cis-regulatory variants may be the target of the detected positive selection. Besides, we found that most of the significant SNPs (21 out of 23) were fixed in the four high-altitude sheep individuals but still segregated in the eight lowland Asian sheep individuals (Fig. 4A), further indicating the *FGF-7* locus was under directional selection.

We then extracted the conservation score for 23 amniota vertebrates of these SNPs for the *FGF-7* loci from Ensembl sheep genome (Oar_v3.1) to align with the above 21 SNPs showing significant genetic differentiation between the high and lowland sheep. Interestingly, we found that one SNP, located at the position of chromosome 7: 57,843,681, was overlapped with one extremely evolutionary conserved element (vertical dash line in Fig. 4A). The substitution site was 2,003 bp upstream of the start codon of *FGF-7* gene and showed 100% sequence similarity among 18 mammal species except for the mutation present in the high-altitude sheep (Fig. 4B), indicating the functional significance of this mutation, probably in the transcriptional regulation of the *FGF-7* gene by disrupting the cis-regulatory element.

### Validation of the upstream regulatory substitution in *FGF-7* gene

Examination of sheep genome sequence was based on a limited number of individuals, prompting analysis of an expanded collection of animals using Sanger sequencing to determine genotype. The results showed that the allele “A” of the SNPs in the regulatory region of *FGF-7* was fixed in Bhyanglung (100%, n = 15) with intermediate allele frequency in Baruwal (56.45%, n = 31), and rare in lowland populations (Kage, 10.71%, n = 28; Lampuchhre, 2.5%, n = 20) (Fig. 4C). The number of individuals is still low; however the frequency of the mutant allele corresponded closely with the elevated altitude, supporting that the T- > A substitution is the target SNP at the *FGF-7* locus for high-altitude adaptation.

The functions of this mutation in the regulatory region were then assessed by a bioinformatics analysis using TFBIND (http://tfbind.hgc.jp/), which detected a remarkable change in the putative transcription factor (TF) binding sites caused by this substitution (Supplementary Table S12). For example, the predominant “A” allele of this SNP in high-altitude sheep may create a putative binding site for a few TFs such as CCAAT enhancer-binding protein (CEBPB), octamer-binding protein (OCT), Yin Yang-1 (YY1), Interferon regulatory factor (IRF1). In contrast, the mutation may also destroy putative target site for some TFs, such as X-box binding protein 1 (XBP1), the aryl hydrocarbon receptor nuclear translocator (ARNT), and so on.

To confirm the observation based on the TFBIND prediction, an electrophoretic mobility shift assay (EMSA) with nuclear extracts from human alveolar epithelial cells was performed. Interestingly, the results revealed specific DNA-protein interaction, providing support to the bioinformatics prediction (Fig. 4D). The biotin labeled probe with the mutant allele, which is the major allele in high-altitude sheep, showed a gel shift by binding with unknown protein complex and the excess of the cold mutant probe can successfully compete out the complex, indicating the specific interaction between unknown proteins with the mutant sequence. However, the probe with the wild type allele cannot compete out the specific interactions, suggesting the candidate SNP generates a new DNA binding site upstream of *FGF-7* gene for some unknown factors. Thus, the altered DNA-protein interactions were identified for the SNP, located at 2,003 bp upstream of the start codon of *FGF-7* gene, qualifying it as candidate causal SNP that may be contributed to the selective signatures of this gene.

## Discussion

By our genome-wide scan of four major Nepalese sheep breeds combined with other 17 downloaded Asian sheep populations, we identified the major selective sweeps for high-altitude adaptation in sheep at the Himalayas. Based on two distinct high-altitude sheep groups, we then mapped the common selective sweep to the most evident candidate *FGF-7*. Despite no previous association of *FGF-7* gene with high-altitude adaptation, its protection role in lung injury was well established. *FGF-7*, as a member of the fibroblast growth factor family with predominant expression in epithelial cells^34^, increases proliferation, inhibits apoptosis, improves barrier function and supports surfactant production in lung epithelial cells^35^,^36^,^37^. In particular, *FGF-7* expression reduces pulmonary edema, permeability, hypoxia, and epithelial injury in various rodent models^38^,^39^ and human *in vitro* model^40^. Thus, *FGF-7* was considered as an intervention to reduce epithelial injury and improve recovery in the acute respiratory distress syndrome^39^. Furthermore, previous genome-wide association study of large cohorts of subjects identified *FGF-7* as a susceptibility locus for chronic obstructive pulmonary disease^41^. These evidences supported that *FGF-7* could be a good candidate for the prevention of pulmonary injury caused by high-altitude environment, such as high-altitude pulmonary edema (HAPE).

The phylogeographic analyses based on PCA, phylogenetic tree, and LD decay, confirmed that both genetic background and breeding history of the four Nepalese sheep breeds were distinct, specifically in the two high-altitude groups, namely, the Tibetan group consisting of Bhyanglung (also Tibetan and Changthangi), and the Himalayan group consisting of Baruwal (Fig. 2). This observation is also supported by previous conclusion made from biochemical study^5^. The two distinct high-altitude sheep groups provided a great opportunity to compare the adaptive mechanism within domestic sheep. Concerning the different genetic background and breeding histories in the Tibetan and Himalayan groups, we can conclude that the *FGF-7* locus identified by the *d*i analysis were more likely attributed to the directional selection rather than the genetic drift or formation of breeds.

Interestingly, by analyzing the re-sequencing data of 12 sheep individuals, we identified a putative regulatory substitution upstream of *FGF-7* is probably the target of the high-altitude selection. First, the SNP displayed a marked allele frequency divergence between the analyzed high- and low-altitude sheep individuals (Fig. 4A). Second, the SNP occurred in an extremely conserved site (Fig. 4B). Further validation in larger populations showed the frequency of the mutant allele corresponded closely with the elevated altitude (Fig. 4C). In addition, our TFBIND prediction indicated this substitution may alter the specific binding sites for TFs in the promoter region of *FGF-7* gene (Supplementary Table S12). A gel shift analysis with the nuclear extracts from human alveolar epithelial cell further supported the prediction (Fig. 4D). Together, the regulatory substitution upstream of *FGF-7* appears to contribute to the signal of selection at the *FGF-7* locus by regulating its expression.

Although the convergent evolution between different species was reported in Tibetans^14^,^16^,^17^, Tibetan mastiff^21^,^22^,^23^, Tibetan grey wolf^20^ and Tibetan goat^28^ with *EPAS1* gene as the common selected locus, yet most high-altitude species have unique adaptive mechanisms. Our genomic analysis revealed the *FGF-7* gene exhibited the strongest genetic differentiation between sheep at Himalayas and their Asian neighbors, suggesting a potential involvement of this gene in high-altitude adaptation. Further examination of the divergence variants in other populations such as Chinese lowland populations should enhance our understanding of the evolution of this gene. We thus hypothesized that *FGF-7* gene potentially enhances lung function by regulating its expression level in high-altitude sheep through altering its binding of specific TFs. Follow-up experimental studies will be needed to validate the hypothesized roles. Interestingly, no evidence has been found that the selective signature of *FGF-7* gene was shared by other high-altitude species. Therefore, our findings provided a potential novel molecular mechanism for the genetic adaptation to high-altitude environments.

## Materials and Methods

### Samples

**Figure 1:**
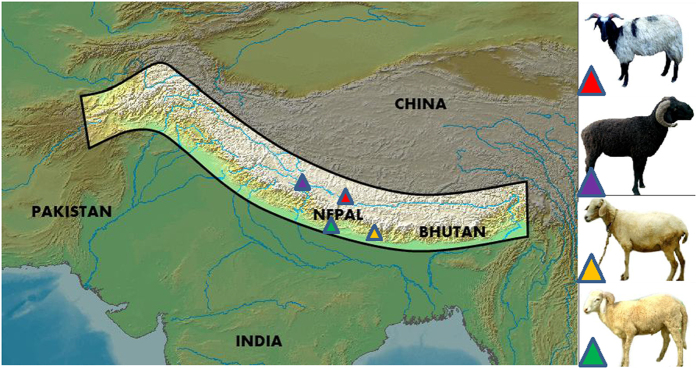
Geographical coverage of Tibetan plateau and the sampling locations for Nepalese sheep breeds in the Himalayas. Indigenous sheep of Nepal (a) Bhyanglung 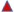, (b) Baruwal 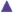, (c) Kage 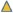 and (d) Lampuchhre 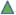. The schematic map used here was adapted from https://en.wikipedia.org/wiki/File:Himalayas_Map.png.

**Figure 2:**
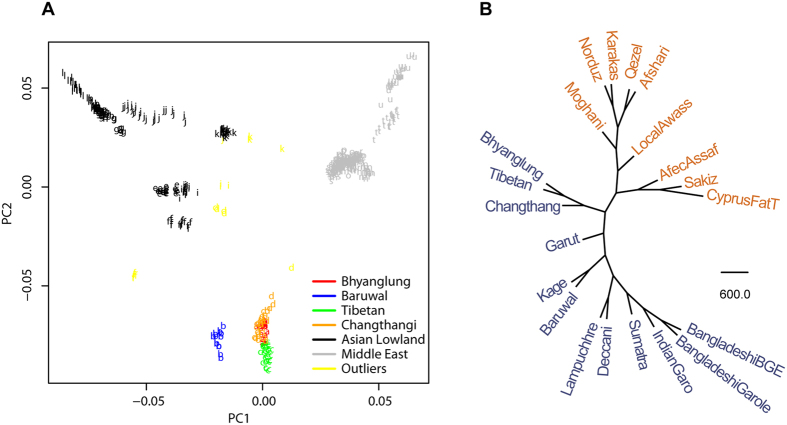
Phylogenetic analysis of 12 Asian and nine Middle East sheep breeds based on 36,711 autosomal SNPs identified by LD-based SNP pruning. (**A**) Principle component analysis of 550 individuals. The first (PC1) and second (PC2) principal components are shown. Each letter presented a breed. a: Bhyanglung; b: Baruwal; c: Tibetan; d: Changthangi; e: Lampuchhre; f: Kage; g: BangladeshiBGE; h: BangladeshiGarole; i: Deccani; j: Sumatra; k: Garut; l: IndianGarole; m: Qezel; n: AfecAssaf; o: Afshari; p: LocalAwassi; q: Karakas; r: Norduz; s: Moghani; t: Sakiz; u: CyprusFatTail. (**B**) Unrooted neighbor-joining phylogenetic tree of sheep breeds. The 12 sheep breeds labeled with blue color are Asian sheep and the nine breeds labeled with brown color are Middle East sheep.

**Figure 3:**
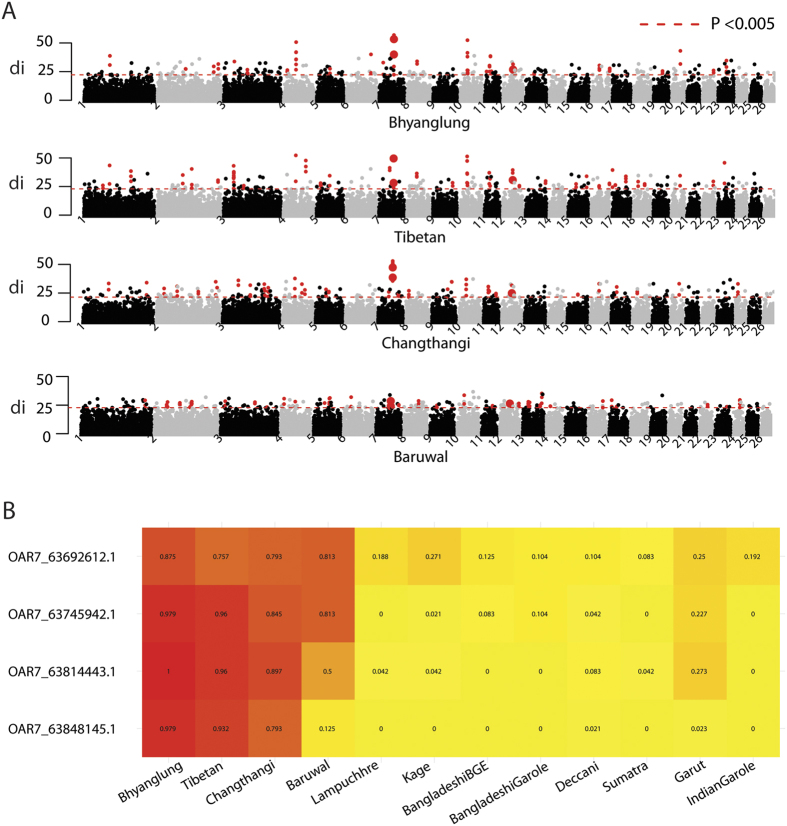
Identification of directional selection for high-altitude adaptation. (**A**) Manhattan plot of genome-wide distribution of *di* values for each of the four high-altitude sheep breeds. Red dots represent significant SNPs within merged regions. The larger red dots indicate common significant SNPs shared by the four breeds, and the threshold indicating signature of selection is denoted with a dashed red line. (**B**) A heat map of frequencies of major allele in high-altitude sheep of the top SNP loci for each tested populations.

**Figure 4:**
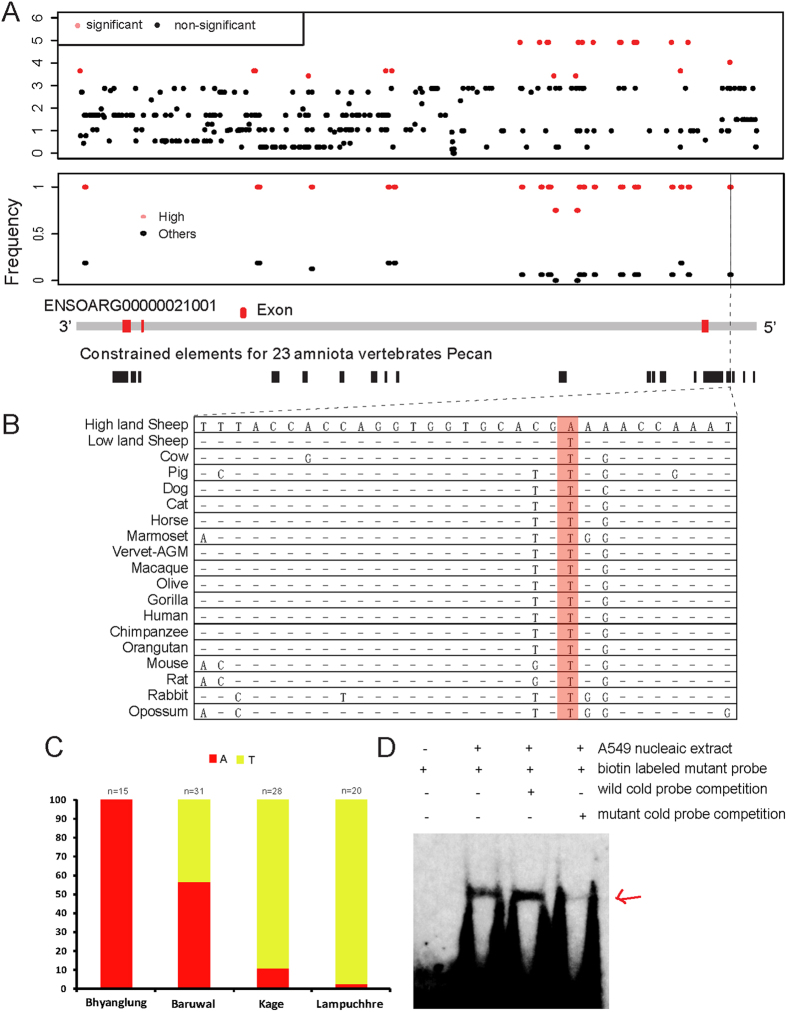
Details of the *FGF-7* locus. (**A**) The distribution of −log_10_(P value) from the Fisher’s exact test for all 329 SNPs and the frequency of the 23 significant SNPs (major allele in high-altitude sheep) in high-altitude populations (red) and in other populations (black). The 23 significant SNPs (P value < 0.001) are indicated in red, while the non-significant ones in black. The ensemble gene model of *FGF-7* gene, with the exons colored in red and the intron/intergenic regions are colored in grey, were shown together with the constrained elements for 23 amniota vertebrates Pecan (black bar). (**B**) Sequence alignment of the constrained element containing the interesting upstream SNP among 18 vertebrates. The SNP position among all 18 vertebrates is shaded in red. “–” indicates the same base with the sequence of high-land sheep. (**C**) Allele frequency of the upstream mutation in *FGF-7* of the four Nepalese sheep breeds. (**D**) EMSA with nuclear extracts from human alveolar epithelial cell line A549 are showed for the candidate SNP. Allele-specific gel shifts are indicated by arrows. Cold probes at 100-fold excess were used to verify specific DNA-protein interactions.

Blood samples from a total of 96 individuals, 24 each for four Nepalese sheep breeds (Bhyanglung, Baruwal, Kage and Lampuchhre) located at different ranges of altitudes were collected from multiple flocks in order to capture representative samples for the within-breed genetic diversity (Fig. 1 and Table 1). We used verbal evidences from the animal owners and their neighbors to make sure the sampled sheep were unrelated at least to the level of grandparents. Each sheep was carefully confirmed to match the phenotypic characteristics of that breed. Genomic DNA was extracted from whole blood using the standard phenol/chloroform extraction protocol.

All the animal experimental procedures were approved by and performed according to the guidelines for the care and use of experimental animals established by the Ministry of Agriculture of People’s Republic of China and Institute of Animal Science, Chinese Academy of Agricultural Sciences.

### Genotyping and data quality control

All genomic DNA samples from 96 sheep were genotyped using the Illumina *Ovine* SNP50 beadchip array, which included 59,454 SNPs, according to the manufacturer’s protocols. We obtained the genotyped SNP data of 71 breeds of sheep (n = 2,957) from the Sheep Genomic Consortium project (ISGC, http://www.sheephapmap.org/hapmap.php)^42^. The downloaded dataset was also generated by Illumina Ovine SNP50 beadchip array and thus readily comparable to our data. Subsequently, the SNP data of the four Nepalese sheep populations in our study were merged with the 17 downloaded datasets including eight Asian (n = 210) and nine Middle East (n = 244) populations. Finally, we generated a 550- individual dataset containing four high-altitude and 17 low-altitude sheep breeds (Table 1), with 49,034 overlapping SNPs. SNPs that failed in any of the following conditions were removed using PLINK v2.05^43^: (1) with call rate <0.90; (2) with minor allele frequency (MAF) < 0.05; (3) with missing genotype data >0.10; individual with more than 10% missing genotype data; (4) not included in the latest reference assembly of the sheep genome Oar_v3.1; (5) located on chromosomes X and Y were removed. After filtering, a total of 45,184 autosomal SNPs were remained for further analysis. None of the samples were excluded.

### Phylogenetic analysis

A pruned data set of 550 sheep containing 36,711 SNPs which excluded SNPs in LD (PLINK, –indep-pairwise 50 5 0.2) were used to investigate the genetic structure. PCA were performed with the ACTG software^44^ and the individuals outside of their expected population clusters were excluded from further analysis. The neighbor-joining tree was constructed using PHYLIP 3.68 software^45^ on the basis of the genome-wide allele frequency data. The pairwise r^2^ values within each populations were calculated with parameter –r2 –ld-window 99999 –ld-window-r2 0 in PLINK^43^ to compare LD patterns among breeds.

### Detection of selective signals in high-altitude sheep

To identify the genomic selective signatures related to altitude adaptation in highland breeds (Bhyanglung, Baruwal, Tibetan and Changthangi), we performed four separated analyses for these breeds by using a total of 43,835 SNPs (MAF < 0.05 among Asian populations). Each breed was compared with other eight lowland breeds found in Asia based on the results of the genetic structure. The unbiased estimate of pairwise *F*_ST_ as described by Weir *et al*.^10^ was calculated using Genepop 4.3 software^46^. Then the *d*_*i*_ statistic at each SNP marker for each high-altitude population, which is particularly well suited for detecting lineage-specific selective events, was calculated for each SNP to retrieve candidate SNPs under selection as described by Akey *et al*.^11^. Specifically, consider *i* high-altitude populations and denote the expected value and standard deviation of F_ST_ between i and *j*th subpopulation as 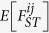 and 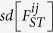, respectively. The di was calculated by the following equation:


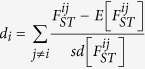


The empirical p-value for *di* (P_E_) was calculated as the proportion of di values that were greater than or equal to the observed di value as following formula:


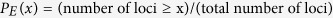


The significance of *d*_*i*_ value was determined as ones falling to 99.5% percentile of the empirical distribution (P_E_ < 0.005). To account for stochasticity in single SNPs, we then clustered all significant SNPs within 500 kb of each other into single highly differentiated reigons.

### *FGF-7* mutation analysis

The downloaded genomic variations stored in Variant Call Format (VCF) files for two Tibetan, two Changthangi and eight other Asian sheep (Supplementary Table S5) were generated by ISGC through the Illumina Hiseq platform and further analyzed by the NextGen of Ensembl Projects (http://projects.ensembl.org/nextgen/). The sequence mutations of the most promising candidate gene, *FGF-7* (chr7: 57,774,972 - 57,846,735) spanning 5 kb upstream of the initial codon or downstream of the terminal codon of the gene) were then extracted by using a custom Perl script. Fisher’s exact test was carried out by testing each position against their population grouping. SNPs with P value < 0.001 were considered as the interesting SNPs. The levels of conservation of these SNPs (Constrained elements/score for 23 amniota vertebrates Pecan), measured by Genomic Evolutionary Rate Profiling (GERP) score^47^, were extracted from Ensembl genome browser for sheep and overlapped with the significant SNPs at the selective sweeps.

Polymerase chain reaction primers (Forward: 5′-CAGTGGTCATGAGAGGGAGC-3′, chr7: 57,842,652 - 57,842,671; Reverse: 5′-GAAGGGTCATCCCCTTCTGC-3′, chr7: 57,843,870 - 57,843,851) were designed for sequencing the promising causal SNP in the upstream of *FGF-7*. After PCR amplification, Sanger’s sequencing technology was employed to genotype the SNP in the additional Nepalese sheep samples from the different altitudes, including 15 Bhyanglung, 31 Baruwal, 28 Kage, and 20 Lampuchhre sheep individuals.

### EMSA

A549 cells were obtained from JK green and were propagated in Dulbecco’s Modified Eagle’s Medium (DMEM) supplemented with 10% heat-inactivated fetal bovine serum and penicillin (0.2 U/ml)/streptomycin (0.2 μg/ml)/L-glutamine (0.2 μg/ml) (Gibco). The nuclear proteins from A549 cells were extracted according to instructions of nuclear and cytoplasmic extraction reagents kit (Beyotime, Beijing, China).

The SNP upstream of sheep *FGF-7* was functionally assessed using EMSA to reveal its potential to affect DNA-protein interaction. The probes used were as follows: *FGF-7* wt, 5′-aggtggtgcacgTaaaccaa-3′; *FGF-7* mut, 5′-aggtggtgcacgAaaaccaa-3′. The probes were purchased 5′-Biotin labelled from Invitrogen. Single-stranded complementary oligos were annealed in 1X NEB2 buffer (New England Biolabs) at 2 min at each degree from 95 °C to 25 °C to produce double-stranded probes. A total of 5 μg A549 nuclear extracts was preincubated on ice for 20 min in binding buffer (kit specific binding buffer with supplements: 30.1 mM KCl, 2 mM MgCl_2_, 0.1 mM EDTA, 0.063% NP-40, 7.5% Glycerol, 1 μg/ml Poly (dI·dC)). Competition reactions were supplemented with 20 pmol (100-fold molar excess) unlabeled ds-oligonucleotide. After the addition of 200 fmol 5′-Biotin labeled ds-oligonucleotide, reactions were incubated at RT for 30 min. The protein–DNA complexes were separated on a 6% polyacrylamide gel (JKgreen) run in 0.5 × TBE at 100 V for 2:30 h in RT. Transfer to IMMOBILON NY + INYC00010 nylon membranes (Millipore) was carried out in 0.5 × TBE at 300 mA, 4 °C for 30 min. The DNA was crosslinked for 60 sec on a transilluminator with 254 nm bulbs and 120 mJ/cm^2^, and blocked with the blocking reagent. The membrane was then detected using streptavidin-horseradish peroxidase conjugate and ECL chemiluminescent detection kit (JKgreen).

## Additional Information

**How to cite this article**: Gorkhali, N. A. *et al*. Genomic analysis identified a potential novel molecular mechanism for high-altitude adaptation in sheep at the Himalayas. *Sci. Rep.* **6**, 29963; doi: 10.1038/srep29963 (2016).

## Supplementary Material

Supplementary Information

## Figures and Tables

**Table 1:** Asian and Middle East sheep (breeds) according to different altitude locations.

Population	Number	Origin	Sampling
**High-altitude sheep**			
Bhyanglung	24	Nepal, Asian	This study
Baruwal	24	Nepal, Asian	This study
Tibetan	37	China, Asian	Downloaded
Changthangi	29	India, Asian	Downloaded
**Low-altitude sheep**			
Lampuchhre	24	Nepal, Asian	This study
Kage	24	Nepal, Asian	This study
BangladeshiBGE	24	Bangladesh, Asia	Downloaded
BangladeshiGarole	24	Bangladesh, Asia	Downloaded
Deccani	24	India, Asia	Downloaded
Sumatra	24	Indonesia, Asia	Downloaded
Garut	22	Indonesia, Asia	Downloaded
IndianGarole	26	India, Asia	Downloaded
Qezel	35	Middle East	Downloaded
AfecAssaf	24	Middle East	Downloaded
Afshari	37	Middle East	Downloaded
LocalAwassi	24	Middle East	Downloaded
Karakas	18	Middle East	Downloaded
Norduz	20	Middle East	Downloaded
Moghani	34	Middle East	Downloaded
Sakiz	22	Middle East	Downloaded
CyprusFatTail	30	Middle East	Downloaded

**Table 2:** Top 10 SNPs with highest *d*_*i*_ values in the three high-altitude breeds belonging to the Tibetan group.

Bhyanglung		Tibetan		Changthangi	
Chr	Position	Chr	Position	Chr	Position
7	OAR7_63848145.1	4	OAR4_51489408.1	7	OAR7_63848145.1
7	OAR7_63814443.1	10	OAR10_29511510.1	7	OAR7_63814443.1
7	OAR7_63745942.1	7	OAR7_63848145.1	7	OAR7_63745942.1
10	OAR10_29511510.1	7	OAR7_63814443.1	7	OAR7_63692612.1
4	OAR4_51489408.1	7	OAR7_63745942.1	4	OAR4_51489408.1
20	OAR20_37437726.1	4	s11336.1	10	OAR10_57152217.1
4	OAR4_51346813.1	10	OAR10_29469450.1	10	OAR10_29511510.1
4	OAR4_51241289.1	23	OAR23_27112379.1	23	s31567.1
10	OAR10_29538398.1	1	OAR1_107504871.1	3	s25321.1
7	OAR7_63692612.1	4	OAR4_51489408.1	7	OAR7_63848145.1

The four common SNPs on chromosome 7 in the Tibetan group are colored in red.
