# Supplementary material for: Genomic analysis identified a potential novel molecular mechanism for high-altitude adaptation in sheep at the Himalayas
Source: Sci Rep. 2016 Jul 22;6:29963. doi: 10.1038/srep29963 (PMC4995607; doi:10.1038/srep29963)
Supplement: Supplementary Information [file srep29963-s1.pdf]

Genomic analysis identified a potential novel molecular mechanism for high-altitude adaptation in sheep at the Himalayas

Neena Amatya Gorkhali[1,2,3,+], Kunzhe Dong[1,3,+], Min Yang[1,3], Shen Song[1,3,4], Adiljian Kader[1,3], Bhola Shankar Shrestha[2], Xiaohong He[1,3], Qianjun Zhao[1,3], Yabin Pu[1,3], Xiangchen Li[1,3], James Kijas[6], Weijun Guan[1,3], Jianlin Han[1,3.5], Lin Jiang[1,3,*], Yuehui Ma[1,3,*]

[1]Institute of Animal Sciences, Chinese Academy of Agricultural Sciences (CAAS), No. 2 Yuanmingyuan West Road, Beijing 100193, China;

[2]Animal Breeding Division, National Animal Science Institute, Nepal Agriculture Research Council (NARC), Kathmandu, Nepal;

[3]CAAS-ILRI Joint Laboratory on Livestock and Forage Genetic Resources, Institute of Animal Science, Chinese Academy of Agricultural Sciences (CAAS), Beijing 100193, China;

[4]Department of Animal Genetics and Breeding, China Agricultural University, Beijing 100094, P. R. China;

[5]International Livestock Research Institute (ILRI), P.O. Box 30709, Nairobi 00100, Kenya

[6]CSIRO Livestock Industries, St Lucia, Brisbane, Qld, Australia

[*]Correspondence author: Lin Jiang: jianglin@caas.cn; Yuehui Ma: mayuehui@caas.cn

[+]These authors contributed equally to this work

Supplementary Figures S1 and S2

Fig. S1. Decline in genome-wide linkage disequilibrium (LD) across and within breeds. Genome-wide LD was estimated both within and across breeds, by calculating r^2 values between all pairs of SNPs with inter-SNP distances of less than 1 Mb.

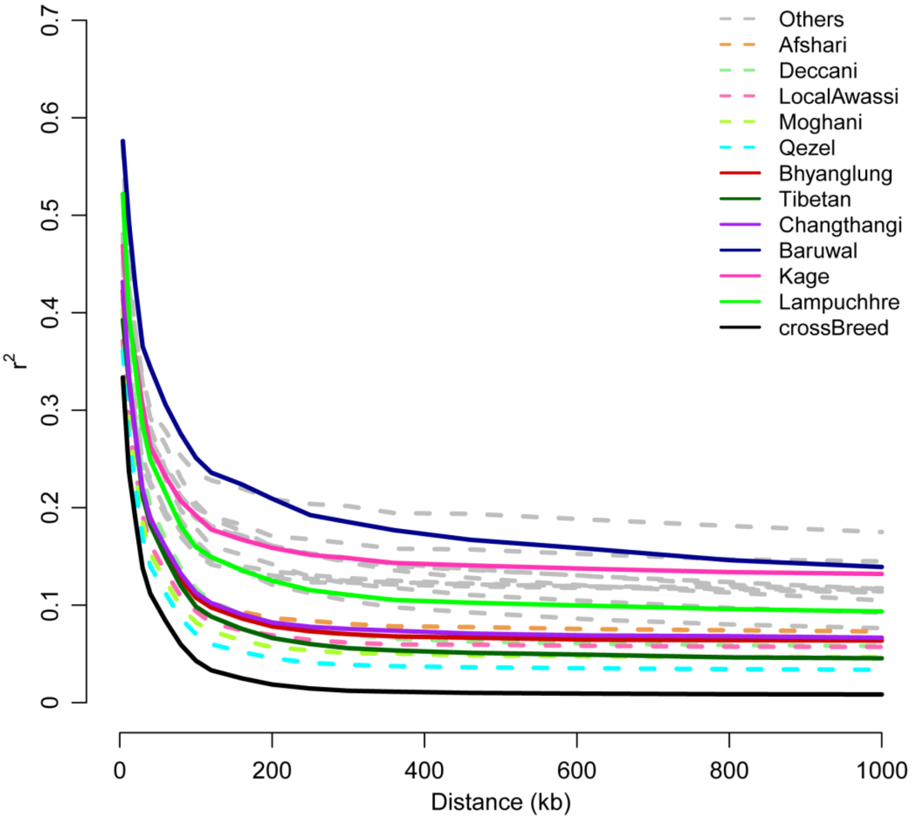

Fig. S2. Comparison of significant SNPs identified in the four high-altitude sheep breeds.

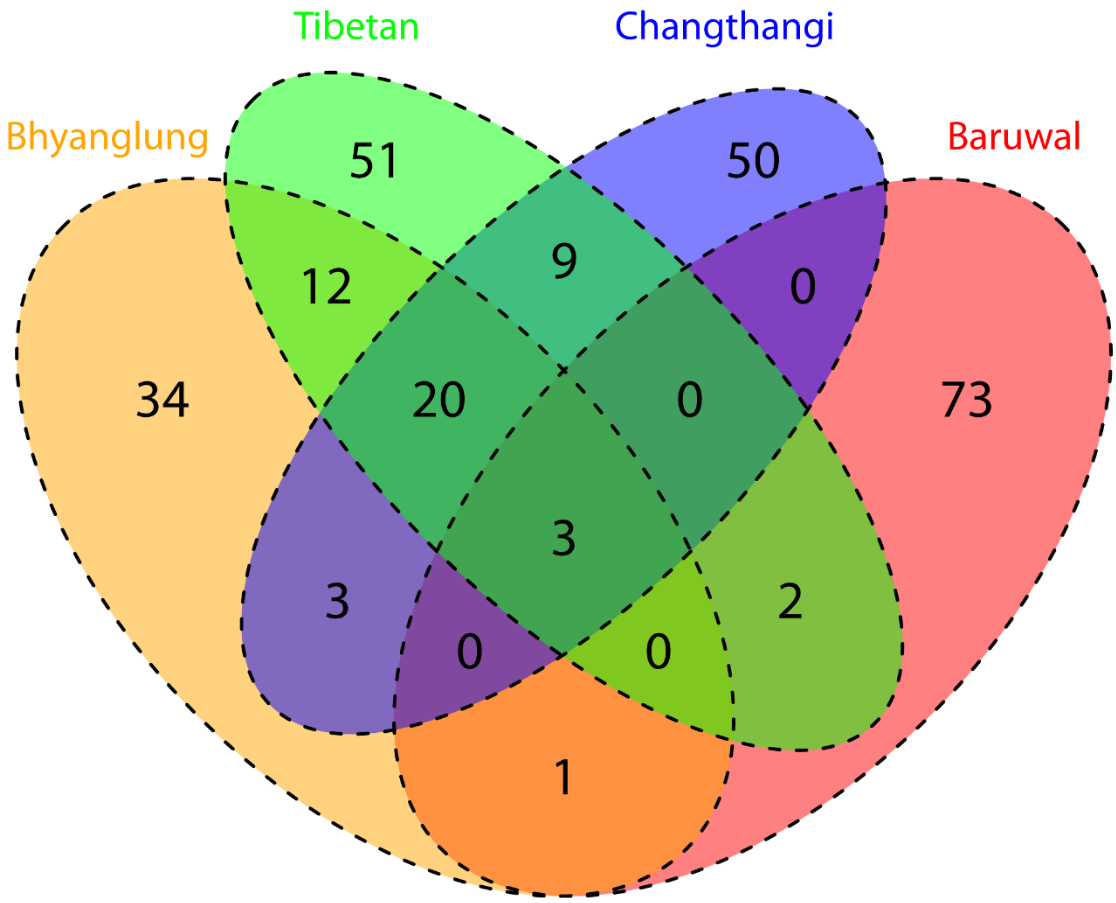

Supplementary Tables S1-S12

Table S1. Genetic differentiation (F_{ST} values) between high and low-altitude sheep breeds

High / Low	Bhyanglung	Tibetan	Changthangi	Baruwal
BangladeshiBGE	0.1098	0.1187	0.108	0.1739
BangladeshiGarole	0.1175	0.1263	0.1165	0.1841
Deccani	0.0809	0.0914	0.0805	0.1479
Garut	0.1119	0.1214	0.1112	0.1835
IndianGarole	0.1347	0.1426	0.133	0.1991
Kage	0.089	0.0988	0.0894	0.1482
Lampuchhre	0.1124	0.1215	0.1114	0.1775
Sumatra	0.125	0.1333	0.1236	0.1907

Table S2. Significant SNPs identified in Bhyanglung sheep

Chr	SNP	Position	Fst between Bhyanglung and								Di
			BanglBGE	BanglGarole	Deccani	Garut	IndianGarole	Kage	Lampuchhre	Sumatra	
1	OAR1_15762141.1	15765887	0.59	0.71	0.26	0.56	0.30	0.38	0.67	0.12	22.02
1	OAR1_61237925.1	58019251	0.33	0.38	0.46	0.59	0.68	0.19	0.79	0.22	22.20
1	s43910.1	67062156	0.41	0.70	0.11	0.66	0.60	0.39	0.24	0.60	22.24
1	OAR1_72984979.1	68457284	0.58	0.62	0.51	0.46	0.59	0.06	0.58	0.50	24.15
1	OAR1_107239370.1	99931717	0.55	0.69	0.50	0.46	0.68	0.42	0.69	0.63	29.80
1	OAR1_107504871.1	100185774	0.72	0.72	0.71	0.70	0.73	0.69	0.72	0.56	37.45
1	s31815.1	106855010	0.61	0.50	0.63	0.13	0.38	0.49	0.37	0.38	21.74
1	s57575.1	182640823	0.30	0.59	0.34	0.35	0.50	0.63	0.25	0.64	22.16
1	OAR1_197916874.1	183420073	0.42	0.30	0.60	0.42	0.45	0.52	0.37	0.68	23.72
1	OAR1_199252158.1	184627258	0.58	0.64	0.48	0.37	0.74	0.74	0.50	0.76	31.44
1	OAR1_232102593.1	215086209	0.51	0.64	0.57	0.30	0.38	0.26	0.38	0.48	21.70
1	s18320.1	243069237	0.73	0.83	0.31	0.68	0.84	-0.07	0.11	0.50	23.59
1	OAR1_268122939.1	248275678	0.87	0.74	0.59	0.24	0.56	0.24	0.51	0.47	26.92
2	OAR2_1640701.1	2808512	0.69	0.61	0.44	0.69	0.57	0.56	0.39	0.81	30.94
2	s00336.1	10805401	0.29	0.37	0.57	0.46	0.65	0.16	0.78	0.46	23.03
2	s28736.1	13932966	0.43	0.41	0.56	0.17	0.49	0.17	0.68	0.67	21.88
2	s13817.1	42253856	0.66	0.66	0.34	0.10	0.67	0.26	0.46	0.55	22.39
2	OAR2_56383269.1	52536647	0.45	0.64	0.75	0.08	0.16	0.37	0.57	0.48	21.96
2	OAR2_64231337.1	59762935	0.36	0.30	0.20	0.64	0.50	0.35	0.85	0.68	23.75
2	OAR2_71530687.1	66963261	0.58	0.65	0.55	0.58	0.66	0.36	0.61	0.27	27.16
2	s62463.1	72644072	0.49	0.73	0.64	0.63	0.77	0.16	0.36	0.70	28.54
2	s48950.1	88900607	0.81	0.67	0.49	0.13	0.71	0.69	0.62	0.81	32.30
2	OAR2_107945014.1	100364649	0.66	0.69	0.27	0.59	0.45	0.35	0.17	0.71	23.82

2	OAR2_118498709.1	110291246	0.44	0.58	0.41	0.62	0.62	0.53	0.63	0.35	26.51
2	s64230.1	110743186	0.39	0.60	0.69	0.42	0.43	0.59	0.65	0.30	26.40
2	OAR2_143916009.1	135304995	0.53	0.63	0.60	0.64	0.67	0.54	0.43	0.66	30.51
2	OAR2_161368305.1	152215629	0.47	0.64	0.23	0.69	0.63	0.28	0.16	0.69	22.86
2	OAR2_173495274.1	163845372	0.82	0.85	0.30	0.68	0.78	0.73	0.51	0.65	34.94
2	s33598.1	217834549	0.70	0.70	0.27	0.35	0.61	0.58	0.33	0.14	22.66
2	s27557.1	220053519	0.58	0.81	0.48	0.59	0.71	0.24	0.64	0.46	28.66
2	OAR2_232672067.1	220103004	0.19	0.50	0.43	0.66	0.12	0.47	0.77	0.67	23.85
2	s41966.1	237813990	0.45	0.77	0.75	0.08	0.68	0.60	0.61	0.72	30.57
2	OAR2_251166495.1	237822440	0.38	0.72	0.69	0.03	0.62	0.52	0.54	0.66	26.58
2	s10664.1	244129054	0.50	0.57	0.34	0.29	0.66	0.21	0.36	0.72	21.92
3	OAR3_3313767.1	3473658	0.65	0.49	0.71	0.77	0.55	0.30	0.24	0.53	27.23
3	s61491.1	9428422	0.38	0.76	0.33	0.67	0.88	0.55	0.77	0.55	31.53
3	OAR3_44467884.1	41551185	0.60	0.34	0.22	0.57	0.26	0.55	0.53	0.63	23.03
3	s72391.1	41793029	0.77	0.63	0.44	0.58	0.73	0.38	0.77	0.74	32.75
3	OAR3_45498973.1	42501688	0.53	0.59	0.42	0.28	0.47	0.53	0.59	0.24	22.80
3	OAR3_60234650.1	56972116	0.63	0.42	0.44	0.84	0.51	0.28	0.07	0.82	24.97
3	OAR3_66317995.1	62704565	0.78	0.40	0.25	0.58	0.42	0.35	0.49	0.45	22.89
3	OAR3_68631966.1	64971662	0.63	0.47	0.53	0.58	0.73	0.48	0.47	0.30	26.81
3	s17646.1	90772017	0.21	0.32	0.85	0.45	0.44	0.01	0.75	0.53	22.16
3	s29393.1	92401501	0.45	0.47	0.41	0.29	0.62	0.69	0.43	0.74	25.82
3	OAR3_98186564.1	92497164	0.28	0.58	0.51	0.39	0.69	0.40	0.36	0.54	23.19
3	s67952.1	93522006	0.17	0.55	0.44	0.83	0.50	0.38	0.38	0.41	22.54
3	s64982.1	93725974	0.87	0.68	0.36	0.21	0.34	0.32	0.38	0.54	22.81
3	OAR3_119041340.1	111746572	0.51	0.60	0.25	0.47	0.67	0.48	0.22	0.36	21.65
3	OAR3_135295238.1	126604459	0.53	0.61	0.59	0.58	0.83	0.15	0.36	0.50	26.04

3	s33601.1	133314721	0.46	0.32	0.54	0.34	0.45	0.55	0.52	0.60	23.78
3	OAR3_150785833.1	140970538	0.69	0.38	0.65	0.68	0.36	0.41	0.77	0.82	31.22
3	OAR3_165009241.1	154213690	0.10	0.07	0.83	0.73	0.01	0.35	0.89	0.63	23.10
3	OAR3_171926505.1	160720849	0.60	0.64	0.54	0.68	0.26	0.46	0.33	0.53	25.81
3	OAR3_173071993.1	161858143	0.66	0.66	0.22	0.36	0.67	0.45	0.49	0.52	25.04
3	s35132.1	163307018	0.70	0.79	0.47	0.65	0.86	0.74	0.25	0.36	31.46
3	s05280.1	178423049	0.72	0.50	0.44	0.24	0.11	0.70	0.69	0.41	24.30
3	s70839.1	186368226	0.48	0.48	0.45	0.40	0.60	0.46	0.48	0.51	24.22
3	s17644.1	213209196	0.74	0.46	0.56	0.27	0.83	0.18	0.53	0.27	23.83
3	s38073.1	218664932	0.60	0.44	0.37	0.13	0.66	0.38	0.53	0.58	22.67
3	s50255.1	220327385	0.38	0.41	0.59	0.55	0.65	0.75	0.87	0.00	27.47
4	OAR4_14093754.1	13881724	0.51	0.62	0.55	0.25	0.63	0.28	0.62	0.43	24.22
4	OAR4_14572146.1	14355529	0.61	0.53	0.36	0.43	0.46	0.31	0.66	0.71	25.51
4	OAR4_18596826.1	18345021	0.62	0.62	0.41	0.29	0.26	0.49	0.33	0.62	22.65
4	OAR4_51020419.1	48201750	0.35	0.49	0.54	0.46	0.59	0.55	0.37	0.73	25.82
4	s32219.1	48304254	0.55	0.38	0.51	0.93	0.34	0.59	0.89	0.98	34.36
4	OAR4_51241289.1	48423060	0.79	0.57	0.71	0.90	0.40	0.65	0.94	0.98	40.43
4	OAR4_51315739.1	48500494	0.55	0.33	0.57	0.79	0.25	0.42	0.84	0.89	30.27
4	OAR4_51346813.1	48531186	0.77	0.56	0.80	0.77	0.75	0.59	0.87	0.87	40.62
4	OAR4_51441757.1	48627750	0.68	0.68	0.62	0.54	0.69	0.64	0.68	0.68	34.59
4	OAR4_51489408.1	48669962	0.89	0.82	0.88	0.82	0.92	0.90	0.92	0.91	48.98
4	OAR4_80605717.1	76007878	0.47	0.66	0.42	0.30	0.76	0.65	0.77	0.52	29.23
4	s11336.1	87572495	0.60	0.60	0.58	0.57	0.61	0.42	0.60	0.59	29.55
5	s45523.1	9446854	0.51	0.39	0.65	0.40	0.47	0.34	0.42	0.32	21.79
5	s29567.1	16203101	0.41	0.74	0.75	0.21	0.72	0.22	0.68	0.07	23.96
5	OAR5_19894072.1	17340888	0.52	0.49	0.34	0.22	0.75	0.39	0.87	0.40	24.64

5	OAR5_35737637.1	32189253	0.61	0.46	0.54	0.04	0.70	0.51	0.57	0.63	25.83
5	OAR5_42039615.1	38259222	0.63	0.54	0.24	0.68	0.33	0.50	0.21	0.59	22.92
5	s23065.1	41357859	0.61	0.75	0.40	0.16	0.54	0.62	0.75	0.60	28.50
5	OAR5_53463172.1	49201835	0.42	0.58	0.75	0.59	0.20	0.59	0.72	0.38	27.90
5	OAR5_55949772.1	51535099	0.60	0.61	0.09	0.62	0.40	0.41	0.34	0.67	22.80
5	OAR5_56001782.1	51587928	0.65	0.55	0.60	0.65	0.51	0.40	0.28	0.52	26.66
5	OAR5_94259136.1	86043483	0.44	0.31	0.26	0.27	0.63	0.63	0.56	0.64	23.10
5	OAR5_108396019.1	99527999	0.57	0.51	0.36	0.60	0.68	0.27	0.51	0.40	24.04
6	DU178311_404.1	36839547	0.29	0.58	0.47	0.52	0.60	0.67	-0.03	0.46	21.96
6	OAR6_49878575.1	44950513	0.54	0.57	0.43	0.50	0.59	0.40	0.19	0.57	23.35
6	s55289.1	46602091	0.85	0.79	0.69	0.55	0.78	0.53	0.53	0.59	35.29
6	s52112.1	89145977	0.56	0.56	0.37	0.44	0.57	0.29	0.59	0.55	24.37
6	s21552.1	94639943	0.70	0.77	0.91	0.25	0.92	0.95	0.45	0.74	38.65
6	OAR6_103985982.1	94749679	0.28	0.11	0.29	0.48	0.73	0.62	0.74	0.49	23.13
6	OAR6_121352193.1	106834499	0.47	0.73	0.26	0.33	0.54	0.32	0.64	0.51	23.18
6	OAR6_125621422.1	110561891	0.50	0.64	0.35	0.53	0.45	0.24	0.40	0.53	22.17
7	s24872.1	15988141	0.46	0.44	0.68	0.70	0.80	0.72	0.32	0.68	31.77
7	s18550.1	15995141	0.69	0.29	0.13	0.66	0.79	0.22	0.37	0.51	21.85
7	OAR7_31901602.1	28210606	0.53	0.69	0.46	0.44	0.63	0.39	0.67	0.45	27.10
7	OAR7_47161420.1	42551974	0.48	0.82	0.60	0.74	0.51	0.85	0.53	0.68	34.89
7	OAR7_50297566.1	45575342	0.79	0.79	0.64	0.66	0.79	0.42	0.50	0.78	35.54
7	OAR7_52060307.1	47264055	0.81	0.70	0.36	0.17	0.60	0.71	0.38	0.50	27.00
7	s15898.1	56115836	0.55	0.55	0.44	0.52	0.56	0.46	0.40	0.55	25.41
7	OAR7_63627909.1	57692469	0.55	0.45	0.62	0.30	0.43	0.73	0.61	0.68	28.48
7	OAR7_63692612.1	57764872	0.71	0.74	0.80	0.55	0.63	0.85	0.63	0.76	38.59
7	OAR7_63745942.1	57816492	0.89	0.87	0.98	0.75	0.98	0.95	0.98	0.98	51.47

7	OAR7_63814443.1	57875478	1.00	1.00	1.00	0.76	1.00	0.97	0.96	0.96	53.62
7	OAR7_63848145.1	57915106	0.98	0.98	0.98	0.98	0.98	0.98	0.98	0.98	54.92
7	OAR7_89553402.1	82197428	0.38	0.31	0.54	0.25	0.65	0.42	0.41	0.76	22.98
8	OAR8_10421816.1	9380786	0.70	0.70	0.28	0.68	0.64	0.24	0.41	0.29	24.31
8	OAR8_14883276.1	13331101	0.74	0.26	0.15	0.56	0.48	0.58	0.45	0.34	21.78
8	OAR8_28235071.1	25787069	0.42	0.29	0.41	0.27	0.68	0.49	0.78	0.40	23.17
8	OAR8_35722542.1	32877217	0.55	0.40	0.22	0.39	0.52	0.41	0.55	0.54	21.67
8	OAR8_41046815.1	38108511	0.75	0.75	0.62	0.69	0.75	0.56	0.24	0.65	32.88
8	OAR8_41067189.1	38129829	0.75	0.75	0.55	0.69	0.75	0.56	0.15	0.52	30.70
8	OAR8_55339073.1	51474008	0.43	0.49	0.14	0.33	0.67	0.58	0.59	0.42	22.03
8	OAR8_67469564.1	62674808	0.34	0.44	0.50	0.53	0.61	0.27	0.64	0.52	23.99
8	OAR8_73347880.1	68365499	0.35	0.39	0.50	0.68	0.45	0.41	0.27	0.51	22.01
9	OAR9_28628261.1	27355734	0.35	0.42	0.38	0.55	0.60	0.56	0.45	0.33	22.54
9	OAR9_57584625.1	54900341	0.26	0.56	0.47	0.21	0.70	0.24	0.66	0.57	22.46
9	s70965.1	57575579	0.34	0.35	0.52	0.21	0.62	0.46	0.56	0.54	22.32
9	OAR9_82428531.1	77756891	0.48	0.79	0.33	0.30	0.36	0.36	0.74	0.84	26.41
10	OAR10_10143630.1	11735682	0.38	0.44	0.64	0.69	0.58	0.06	0.24	0.62	22.32
10	OAR10_18125946.1	19014824	0.58	0.31	0.44	0.36	0.77	0.61	0.27	0.22	21.97
10	OAR10_29092520.1	29054709	0.58	0.47	0.38	0.18	0.50	0.73	0.72	0.43	25.33
10	OAR10_29469450.1	29436435	0.75	0.75	0.73	0.47	0.72	0.67	0.68	0.71	36.81
10	OAR10_29511510.1	29476678	0.92	0.92	0.91	0.91	0.92	0.90	0.92	0.89	50.62
10	OAR10_29538398.1	29502667	0.87	0.93	0.21	0.87	0.55	0.75	0.89	0.93	40.07
10	s18834.1	29806294	0.68	0.68	0.40	0.39	0.50	0.25	0.23	0.64	23.19
10	s68983.1	30618253	0.58	0.34	0.62	0.50	0.47	0.32	0.65	0.21	23.18
10	s12004.1	30674002	0.39	0.29	0.32	0.67	0.71	0.25	0.70	0.38	22.58
10	OAR10_30746533.1	30700853	0.51	0.70	0.65	0.42	0.68	0.09	0.70	0.27	25.30

10	OAR10_49074172.1	48300368	0.55	0.60	0.44	0.15	0.58	0.48	0.76	0.13	23.11
10	OAR10_55185347.1	54046513	0.75	0.55	0.47	0.36	0.73	0.45	0.09	0.43	23.91
10	OAR10_57152217.1	56010798	0.84	0.77	0.68	0.82	0.73	0.38	0.64	0.76	37.54
10	OAR10_61672235.1	60158728	0.40	0.40	0.60	0.58	0.90	0.87	0.50	0.38	30.44
11	s30798.1	7858660	0.79	0.67	0.64	0.15	0.44	0.57	0.37	0.18	24.28
11	s36598.1	7898325	0.85	0.48	0.47	0.55	0.50	0.59	0.22	0.17	24.26
11	OAR11_19632538.1	19223203	0.72	0.59	0.46	0.51	0.73	0.45	0.53	0.56	29.32
11	s17310.1	19385272	0.77	0.64	0.74	0.31	0.11	0.35	0.82	0.60	28.38
11	OAR11_19800228_X.1	19397667	0.73	0.64	0.64	0.21	0.21	0.18	0.70	0.54	24.31
11	OAR11_23673049.1	22780752	0.79	0.79	0.64	0.63	0.80	0.50	0.76	0.69	37.33
11	s41017.1	22814442	0.79	0.79	0.61	0.67	0.80	0.46	0.76	0.69	36.98
11	OAR11_27654920.1	26421905	0.57	0.57	0.24	0.41	0.58	0.53	0.21	0.57	22.51
11	s72626.1	26872280	0.66	0.66	0.56	0.36	0.63	0.09	0.52	0.45	24.52
11	OAR11_35138896.1	32905812	0.67	0.67	0.40	0.28	0.56	0.43	0.70	-0.02	22.98
11	s60757.1	39628891	0.73	0.50	0.26	0.27	0.18	0.54	0.42	0.72	22.19
11	OAR11_43313971.1	40826416	0.57	0.44	0.39	0.45	0.08	0.47	0.60	0.56	22.09
11	s46121.1	42992902	0.93	0.82	0.11	0.27	0.64	0.36	0.00	0.80	23.93
12	OAR12_39493157.1	35320580	0.65	0.60	0.58	0.71	0.83	0.30	0.51	0.81	32.35
12	OAR12_43331187.1	38923821	0.53	0.82	0.60	0.35	0.87	0.49	0.35	0.69	30.45
12	OAR12_43408733.1	39002907	0.64	0.56	0.51	0.28	0.66	0.35	0.30	0.84	25.96
12	s27978.1	45732811	0.51	0.25	0.36	0.81	0.72	0.51	0.27	0.63	25.40
12	s21761.1	47186720	0.25	0.71	0.26	0.18	0.69	0.26	0.71	0.61	22.16
12	OAR12_68075884.1	61642822	0.54	0.64	0.58	0.53	0.62	0.17	0.64	0.27	25.08
12	s62974.1	69228088	0.61	0.48	0.51	0.43	0.47	0.62	0.20	0.79	26.14
13	s14792.1	32586633	0.75	0.50	0.45	0.62	0.44	0.34	0.47	0.11	22.88
13	OAR13_35982442.1	32625552	0.78	0.39	0.41	0.33	0.52	0.42	0.50	0.18	21.82

13	OAR13_38906100.1	35507432	0.59	0.59	0.40	0.79	0.53	0.27	0.32	0.41	24.21
13	OAR13_51387503.1	48147260	0.63	0.41	0.36	0.33	0.69	0.32	0.79	0.72	26.63
13	s14721.1	56428019	0.54	0.63	0.72	0.46	0.48	0.53	0.09	0.51	25.41
13	s11373.1	61563593	0.66	0.77	0.01	0.61	0.67	0.21	0.43	0.74	24.87
13	OAR13_81527927.1	75702438	0.72	0.80	0.18	0.39	0.60	0.35	0.25	0.83	25.40
13	s75919.1	78004271	0.54	0.61	0.43	0.34	0.65	0.31	0.61	0.36	23.81
14	s71975.1	16349731	0.56	0.71	0.41	0.37	0.32	0.70	0.29	0.55	24.68
15	OAR15_13425521.1	13417873	0.74	0.85	0.36	0.16	0.83	0.66	0.74	0.32	30.03
15	OAR15_22514728.1	21594492	0.67	0.56	0.29	0.53	0.25	0.55	0.22	0.49	21.83
15	OAR15_44332191.1	42207222	0.66	0.66	0.56	0.03	0.67	0.62	0.66	0.32	26.91
15	OAR15_80616593.1	74407015	0.50	0.59	0.26	0.55	0.44	0.41	0.18	0.65	21.74
16	s24768.1	4731279	0.55	0.55	0.22	0.39	0.56	0.37	0.48	0.47	21.73
16	OAR16_33317775.1	30655866	0.36	0.74	0.47	0.39	0.68	0.41	0.53	0.71	27.06
16	s59565.1	30667706	0.39	0.66	0.53	0.55	0.57	0.34	0.37	0.65	25.57
16	OAR16_33435828.1	30720540	0.59	0.79	0.57	0.47	0.70	0.22	0.62	0.65	29.47
16	s15191.1	39007570	0.61	0.48	0.62	0.43	0.63	0.52	0.61	0.51	28.59
16	OAR16_69511009.1	63750355	0.43	0.70	0.50	0.35	0.82	0.47	0.41	0.10	23.58
16	s53565.1	70710282	0.85	0.71	0.27	0.08	0.80	0.15	0.53	0.65	24.74
16	s51002.1	70900966	0.69	0.74	0.32	0.74	0.83	0.18	0.45	0.36	26.97
16	s37581.1	70922464	0.69	0.74	0.32	0.61	0.83	0.18	0.45	0.36	26.01
17	OAR17_5338968.1	4822132	0.62	0.62	0.60	0.39	0.63	0.58	0.62	0.50	29.60
17	OAR17_28390642.1	25862600	0.53	0.55	0.75	0.24	0.73	0.48	0.63	0.63	29.64
17	OAR17_55001652.1	50523309	0.70	0.67	0.38	0.43	0.74	0.12	0.21	0.73	24.46
17	OAR17_64119946.1	58691585	0.51	0.65	0.47	0.22	0.47	0.56	0.42	0.26	22.16
18	DU486946_262.1	14116240	0.49	0.56	0.38	0.57	0.61	0.34	0.56	0.59	25.53
18	s10918.1	15573541	0.49	0.53	0.51	0.41	0.54	0.31	0.53	0.45	23.52

18	s31152.1	19342316	0.51	0.45	0.39	0.07	0.94	0.45	0.63	0.41	23.72
18	s12590.1	20040105	0.81	0.58	0.50	0.39	0.79	0.53	0.69	0.43	30.70
18	s53519.1	29717895	0.57	0.39	0.63	0.49	0.32	0.35	0.69	0.43	24.48
18	OAR18_41416894.1	38891583	0.78	0.52	0.29	0.42	0.76	0.49	0.50	0.08	23.90
18	s48176.1	67289143	0.66	0.72	0.67	0.06	0.55	0.45	0.66	0.37	26.51
19	OAR19_4318651.1	4077028	0.61	0.63	0.65	0.61	0.46	0.58	0.20	0.55	27.87
19	OAR19_36413643.1	34817411	0.62	0.33	0.55	0.40	0.69	0.00	0.44	0.71	22.88
19	OAR19_36435815.1	34838172	0.61	0.28	0.48	0.64	0.74	0.08	0.50	0.67	24.77
19	s66398.1	53965311	0.55	0.55	0.40	0.28	0.56	0.24	0.55	0.55	22.48
19	s21513.1	55690132	0.61	0.47	0.18	0.40	0.68	0.61	0.61	0.22	23.43
19	s20082.1	56296821	0.69	0.74	0.35	0.52	0.32	0.31	0.45	0.43	23.70
20	OAR20_9024546_X.1	8893097	0.62	0.62	0.33	0.47	0.53	0.58	0.55	0.50	26.71
20	OAR20_9818283.1	9702838	0.40	0.38	0.56	0.36	0.21	0.66	0.45	0.49	22.09
20	OAR20_14826084.1	14045781	0.45	0.23	0.54	0.36	0.63	0.28	0.56	0.51	21.72
20	s59649.1	15613020	0.38	0.50	0.74	0.03	0.73	0.59	0.45	0.48	24.89
20	OAR20_37437726.1	34168829	0.83	0.96	0.83	0.86	0.72	0.56	0.79	0.56	41.67
20	s39515.1	34256995	0.61	0.72	0.78	0.71	0.48	0.38	0.69	0.28	30.62
20	s61980.1	49930884	0.82	0.38	0.39	0.61	0.58	0.49	0.27	0.24	23.61
21	OAR21_12460436.1	10911345	0.66	0.61	0.38	0.42	0.16	0.49	0.50	0.57	23.76
21	s37379.1	30761604	0.42	0.64	0.51	0.41	0.48	0.31	0.71	0.66	26.21
21	OAR21_47788299.1	43241397	0.55	0.42	0.38	0.68	0.38	0.34	0.17	0.77	22.61
21	s65606.1	46568117	0.33	0.46	0.63	0.62	0.46	0.62	0.67	0.72	29.48
22	OAR22_48326768.1	42969386	0.45	0.56	0.41	0.44	0.57	0.56	0.42	0.59	25.21
22	s31685.1	43182266	0.37	0.70	0.47	0.71	0.26	0.29	0.46	0.49	23.30
23	s69818.1	3075709	0.64	0.61	0.26	0.40	0.63	0.43	0.58	0.60	25.93
23	OAR23_27112379.1	25972928	0.61	0.74	0.55	0.22	0.69	0.66	0.53	0.76	30.95

23	OAR23_33956409.1	32282622	0.18	0.50	0.59	0.55	0.55	0.37	0.68	0.29	23.28
23	OAR23_34033124.1	32354664	0.52	0.70	0.64	0.70	0.76	0.57	0.64	0.54	33.48
23	OAR23_34054014.1	32373968	0.47	0.67	0.45	0.60	0.74	0.39	0.55	0.55	28.16
23	OAR23_34148286.1	32466427	0.41	0.70	0.61	0.60	0.27	0.29	0.50	0.49	24.46
23	OAR23_37161222.1	35205521	0.40	0.36	0.66	0.24	0.46	0.60	0.62	0.32	23.21
23	s17722.1	41141185	0.58	0.53	0.56	0.39	0.30	0.75	0.36	0.24	23.90
23	s31567.1	51126740	0.72	0.50	0.73	0.79	0.68	0.56	0.78	0.27	33.53
23	OAR23_63417651.1	59418377	0.58	0.68	0.44	0.16	0.69	0.21	0.61	0.64	24.85
24	s59452.1	9721533	0.72	0.87	0.40	0.70	0.48	0.25	0.24	0.71	27.58
24	s23797.1	36250095	0.32	0.30	0.51	0.38	0.21	0.74	0.66	0.38	21.99
25	s42703.1	17652415	0.69	0.85	0.53	0.61	0.65	0.41	0.19	0.76	30.35
25	s44878.1	32017122	0.59	0.20	0.77	0.17	0.55	0.18	0.62	0.46	22.04
26	OAR26_17518698.1	14644438	0.74	0.31	0.26	0.46	0.68	0.62	0.47	0.24	23.52
26	s33938.1	38189272	0.53	0.41	0.46	0.45	0.50	0.44	0.45	0.48	23.16

Notes: Significant SNPs located within merged genomic regions are colored “red”.

Table S3. Significant SNPs identified in Tibetan sheep

CHR	SNP	Position	Fst between Tibetan and								Di
			BanglBGE	BanglGarole	Deccani	Garut	IndianGarole	Kage	Lampuchhre	Sumatra	
1	s41913.1	2870378	0.59	0.49	0.58	0.63	0.65	0.54	0.18	0.63	26.24
1	OAR1_19174177.1	19048510	0.71	0.80	0.29	0.54	0.38	0.69	0.25	0.17	22.96
1	OAR1_20180147_X.1	20099767	0.77	0.65	0.33	0.71	0.40	0.54	0.36	0.57	26.36
1	OAR1_23447504.1	23376407	0.69	0.62	0.44	0.53	0.60	0.29	0.20	0.52	23.07
1	OAR1_41497208.1	40190989	0.24	0.73	0.63	0.50	0.49	0.81	0.53	0.64	28.74
1	OAR1_47719852.1	46013940	0.64	0.59	0.35	0.60	0.36	0.47	0.42	0.48	23.29
1	OAR1_51833033.1	49698300	0.49	0.44	0.44	0.65	0.68	0.50	0.53	0.10	22.84
1	OAR1_79060603.1	73928927	0.44	0.54	0.68	0.59	0.52	0.49	0.44	0.42	25.28
1	OAR1_79518018.1	74316221	0.63	0.63	0.35	0.24	0.64	0.49	0.32	0.56	22.85
1	OAR1_107239370.1	99931717	0.52	0.65	0.48	0.43	0.64	0.40	0.65	0.59	26.57
1	OAR1_107504871.1	100185774	0.82	0.82	0.81	0.81	0.83	0.80	0.82	0.70	42.20
1	OAR1_139735768.1	129024743	0.59	0.79	0.44	0.72	0.45	0.58	0.28	0.33	25.42
1	OAR1_150132797.1	139006820	0.68	0.49	0.24	0.37	0.69	0.59	0.33	0.43	22.56
1	s55347.1	183336939	0.57	0.48	0.73	0.79	0.50	0.56	0.64	0.91	32.99
1	OAR1_197916874.1	183420073	0.68	0.57	0.82	0.70	0.69	0.78	0.64	0.87	37.38
1	DU464218_590.1	183575060	0.26	0.63	0.61	0.63	0.70	0.74	0.51	0.55	28.75
1	OAR1_208134575.1	192746782	0.52	0.50	0.15	0.69	0.66	0.41	0.57	0.48	23.46
1	s18797.1	196009122	0.73	0.65	0.58	0.20	0.59	0.65	0.54	0.85	29.98
1	OAR1_268122939.1	248275678	0.95	0.86	0.75	0.45	0.71	0.46	0.67	0.64	35.23
2	OAR2_64231337.1	59762935	0.39	0.33	0.22	0.67	0.54	0.39	0.86	0.71	24.42
2	s48402.1	60634939	0.74	0.41	0.55	0.56	0.60	0.05	0.74	0.61	25.69
2	s62463.1	72644072	0.43	0.65	0.57	0.56	0.69	0.12	0.31	0.63	23.47

2	OAR2_87543957.1	82346323	0.53	0.45	0.62	0.55	0.55	0.19	0.64	0.67	25.38
2	OAR2_107787796.1	100199475	0.66	0.68	0.60	0.59	0.51	0.29	0.25	0.82	26.90
2	OAR2_107945014.1	100364649	0.82	0.84	0.50	0.77	0.65	0.58	0.38	0.85	34.10
2	OAR2_118498709.1	110291246	0.41	0.55	0.38	0.59	0.60	0.50	0.60	0.32	23.61
2	OAR2_142924312.1	134460137	0.51	0.51	0.35	0.55	0.55	0.50	0.57	0.29	22.62
2	OAR2_143916009.1	135304995	0.71	0.78	0.77	0.79	0.81	0.73	0.64	0.80	39.22
2	OAR2_143949087.1	135337045	0.35	0.28	0.70	0.42	0.42	0.27	0.78	0.62	23.04
2	OAR2_144244441.1	135636519	0.62	0.57	0.45	0.46	0.26	0.64	0.60	0.62	25.89
2	OAR2_161368305.1	152215629	0.54	0.68	0.31	0.73	0.67	0.36	0.22	0.72	25.19
2	OAR2_166865423.1	157367793	0.51	0.45	0.58	0.37	0.63	0.49	0.60	0.53	25.29
2	s41966.1	237813990	0.47	0.78	0.75	0.07	0.69	0.61	0.63	0.73	29.66
2	OAR2_251166495.1	237822440	0.44	0.75	0.73	0.05	0.67	0.58	0.60	0.70	28.21
2	s10664.1	244129054	0.55	0.61	0.39	0.33	0.69	0.25	0.41	0.75	23.41
2	OAR2_259153647.1	245206162	0.71	0.64	0.31	0.70	0.70	0.39	0.42	0.09	23.47
2	s49602.1	248590000	0.69	0.62	0.33	0.51	0.21	0.62	0.20	0.78	23.74
3	s61491.1	9428422	0.41	0.77	0.36	0.69	0.86	0.58	0.77	0.57	31.05
3	s08514.1	39851694	0.88	0.80	0.35	0.53	0.49	0.84	0.80	0.32	31.74
3	s05744.1	40080488	0.88	0.86	0.87	0.72	0.88	0.81	0.80	0.55	41.88
3	OAR3_42867624.1	40124759	0.76	0.61	0.35	0.29	0.74	0.56	0.46	0.04	22.64
3	OAR3_42907435.1	40163832	0.81	0.69	0.45	0.84	0.80	0.81	0.29	0.27	31.25
3	s51799.1	40441564	0.67	0.62	0.71	0.75	0.45	0.73	0.53	0.77	33.59
3	OAR3_43377195.1	40534487	0.41	0.46	0.68	0.57	0.41	0.88	0.78	0.22	27.84
3	OAR3_43504420.1	40657958	0.84	0.84	0.70	0.69	0.42	0.71	0.61	0.69	35.53
3	OAR3_43559179_X.1	40713016	0.86	0.69	0.74	0.86	0.66	0.47	0.88	0.54	36.82
3	OAR3_43700480.1	40849521	0.60	0.65	0.71	0.91	0.60	0.70	0.69	0.77	36.37
3	OAR3_43817056.1	40956953	0.62	0.62	0.66	0.48	0.46	0.65	0.79	0.76	32.14

3	OAR3_44467884.1	41551185	0.64	0.42	0.31	0.62	0.35	0.61	0.59	0.67	25.54
3	OAR3_63663119.1	60080759	0.44	0.23	0.57	0.47	0.49	0.56	0.39	0.70	23.11
3	OAR3_74181493.1	70193170	0.24	0.36	0.60	0.77	0.58	0.25	0.52	0.77	24.55
3	OAR3_79206128.1	74982457	0.65	0.32	0.73	0.53	0.31	0.08	0.56	0.59	22.52
3	OAR3_84033269.1	79473981	0.44	0.44	0.34	1.00	0.67	0.23	0.38	0.40	22.91
3	OAR3_84073899.1	79511180	0.44	0.44	0.37	1.00	0.67	0.23	0.64	0.40	25.09
3	OAR3_96268784.1	90654749	0.25	0.44	0.82	0.35	0.45	0.04	0.73	0.71	22.63
3	s64982.1	93725974	0.88	0.71	0.38	0.21	0.36	0.33	0.40	0.57	22.81
3	OAR3_103689521.1	97441687	0.54	0.67	0.58	0.42	0.59	0.45	0.73	0.83	29.99
3	OAR3_133503603.1	125162605	0.61	0.55	0.55	0.39	0.79	0.32	0.50	0.26	23.73
3	OAR3_141010154.1	131915865	0.46	0.60	0.68	0.44	0.83	0.17	0.30	0.52	23.88
3	OAR3_148780075.1	139002376	0.68	0.44	0.36	0.46	0.53	0.48	0.20	0.69	22.73
3	OAR3_152693749.1	142848742	0.60	0.74	0.47	0.73	0.81	0.59	0.56	0.71	32.71
3	OAR3_165009241.1	154213690	0.31	0.27	0.94	0.88	0.19	0.60	0.98	0.80	31.90
3	s13456.1	156283498	0.67	0.63	0.10	0.76	0.69	0.57	0.32	0.22	23.32
3	OAR3_194751219.1	180839858	0.54	0.62	0.52	0.38	0.38	0.14	0.64	0.66	22.95
3	s70839.1	186368226	0.49	0.49	0.46	0.42	0.59	0.47	0.49	0.52	23.56
4	OAR4_14572146.1	14355529	0.76	0.70	0.55	0.62	0.63	0.52	0.80	0.83	34.43
4	s32219.1	48304254	0.49	0.30	0.44	0.86	0.26	0.52	0.84	0.91	28.75
4	OAR4_51315739.1	48500494	0.56	0.34	0.58	0.78	0.25	0.43	0.82	0.86	28.76
4	OAR4_51489408.1	48669962	0.95	0.89	0.95	0.90	0.97	0.96	0.97	0.97	50.74
4	OAR4_80559387.1	75968047	0.29	0.49	0.32	0.45	0.65	0.28	0.67	0.74	22.78
4	s11336.1	87572495	0.89	0.89	0.88	0.88	0.89	0.79	0.89	0.88	46.37
4	OAR4_93024080.1	87602738	0.67	0.67	0.86	0.86	0.73	0.23	0.87	0.87	37.02
4	OAR4_93067608.1	87649810	0.90	0.88	0.68	0.79	0.86	0.58	0.80	0.85	41.32
4	s10721.1	94145887	0.65	0.45	0.50	0.72	0.40	0.42	0.50	0.21	23.23

4	s45195.1	105363106	0.76	0.66	0.66	0.51	0.33	0.20	0.78	0.67	28.37
5	s58621.1	1431071	0.64	0.60	0.78	-0.01	0.86	0.13	0.48	0.54	24.18
5	s56763.1	15912332	0.65	0.77	0.61	0.48	0.68	0.38	0.27	0.33	25.41
5	s29567.1	16203101	0.47	0.75	0.76	0.28	0.73	0.29	0.70	0.12	25.12
5	s07332.1	16209393	0.66	0.61	0.82	0.17	0.54	0.35	0.91	0.29	27.19
5	s38680.1	16520048	0.64	0.53	0.62	0.55	0.60	0.50	0.32	0.47	25.81
5	OAR5_22471776_X.1	19778519	0.32	0.67	0.69	0.39	0.64	0.60	0.23	0.75	26.23
5	s07525.1	19986771	0.66	0.45	0.17	0.60	0.86	0.59	-0.02	0.63	23.24
5	OAR5_25491559.1	22769321	0.72	0.51	0.39	0.02	0.51	0.65	0.51	0.47	22.56
5	OAR5_33499998.1	30419920	0.69	0.54	0.35	0.55	0.76	0.62	0.73	0.13	26.70
5	s39187.1	46272815	0.70	0.57	0.53	0.70	0.61	0.24	0.41	0.36	24.75
5	OAR5_55949772.1	51535099	0.65	0.67	0.12	0.69	0.46	0.49	0.40	0.73	25.12
5	OAR5_56001782.1	51587928	0.76	0.68	0.74	0.77	0.65	0.57	0.43	0.66	33.53
5	OAR5_94259136.1	86043483	0.54	0.42	0.38	0.39	0.70	0.71	0.65	0.71	27.72
6	OAR6_22138611.1	19161699	0.59	0.70	0.80	0.70	0.62	0.56	0.23	0.32	28.27
6	OAR6_64407192.1	58445946	0.64	0.55	0.43	0.12	0.65	0.61	0.58	0.49	24.54
6	OAR6_67376317.1	61138606	0.76	0.62	0.60	0.56	0.86	0.45	0.42	0.77	31.63
6	OAR6_94477596.1	86145569	0.86	0.88	0.14	0.54	0.81	0.67	0.10	0.13	24.73
6	s21552.1	94639943	0.75	0.81	0.93	0.30	0.93	0.96	0.51	0.78	39.21
7	s24872.1	15988141	0.48	0.46	0.69	0.71	0.79	0.72	0.34	0.69	30.75
7	s17550.1	42463209	0.69	0.86	0.68	0.80	0.64	0.87	0.46	0.86	38.07
7	OAR7_47161420.1	42551974	0.63	0.90	0.73	0.84	0.65	0.93	0.67	0.79	40.15
7	OAR7_50297566.1	45575342	0.63	0.63	0.48	0.50	0.63	0.27	0.34	0.62	24.58
7	OAR7_52060307.1	47264055	0.80	0.70	0.34	0.13	0.60	0.70	0.37	0.49	25.04
7	OAR7_63572452.1	57631745	0.42	0.36	0.40	0.20	0.36	0.90	0.93	0.25	23.23
7	OAR7_63692612.1	57764872	0.56	0.58	0.64	0.38	0.47	0.69	0.48	0.60	27.37

7	OAR7_63745942.1	57816492	0.88	0.86	0.95	0.75	0.95	0.92	0.95	0.95	48.12
7	OAR7_63814443.1	57875478	0.95	0.95	0.95	0.72	0.95	0.92	0.91	0.91	48.65
7	OAR7_63848145.1	57915106	0.92	0.92	0.91	0.91	0.92	0.91	0.92	0.92	48.98
7	OAR7_87079540.1	79831939	0.65	0.63	0.56	0.50	0.50	0.55	0.51	0.69	28.54
8	DU317902_536.1	10705915	0.74	0.64	0.61	0.29	0.38	0.44	0.49	0.48	24.81
8	OAR8_22748311.1	20115452	0.62	0.63	0.41	0.54	0.75	0.25	0.27	0.71	24.92
8	OAR8_33527267.1	30812613	0.56	0.65	0.19	0.52	0.51	0.35	0.70	0.65	24.57
8	OAR8_40984723.1	38046068	0.75	0.75	0.65	0.70	0.75	0.59	0.31	0.74	33.17
8	OAR8_41046815.1	38108511	0.79	0.79	0.70	0.75	0.80	0.65	0.35	0.71	35.43
8	OAR8_41067189.1	38129829	0.78	0.78	0.62	0.73	0.78	0.63	0.23	0.59	32.50
8	OAR8_46967818.1	43634777	0.62	0.62	0.47	0.21	0.22	0.71	0.47	0.74	24.82
8	OAR8_55339073.1	51474008	0.55	0.60	0.27	0.46	0.74	0.67	0.68	0.54	27.58
8	OAR8_57382929.1	53493005	0.69	0.60	0.41	0.42	0.40	0.58	0.72	0.59	27.17
8	s37234.1	55901517	0.63	0.58	0.58	0.17	0.69	0.17	0.55	0.48	22.80
8	OAR8_67469564.1	62674808	0.48	0.56	0.61	0.64	0.70	0.42	0.72	0.63	29.48
9	s08530.1	15705043	0.38	0.35	0.70	0.29	0.52	0.58	0.71	0.30	23.32
9	s66058.1	32100676	0.61	0.50	0.44	0.48	0.84	0.58	0.67	0.41	28.01
9	OAR9_64800745_X.1	61661127	0.29	0.54	0.57	0.63	0.72	0.71	0.84	0.53	30.22
9	OAR9_81909760.1	77297358	0.39	0.72	0.36	0.74	0.51	0.31	0.72	0.78	27.53
9	OAR9_82428531.1	77756891	0.48	0.78	0.32	0.29	0.36	0.35	0.73	0.82	24.84
10	OAR10_29092520.1	29054709	0.63	0.54	0.45	0.25	0.56	0.77	0.75	0.50	27.49
10	OAR10_29469450.1	29436435	0.90	0.90	0.90	0.72	0.89	0.87	0.86	0.88	45.99
10	OAR10_29511510.1	29476678	0.93	0.93	0.93	0.93	0.94	0.93	0.93	0.91	49.84
10	OAR10_29538398.1	29502667	0.82	0.88	0.15	0.82	0.52	0.71	0.84	0.88	35.62
10	s18834.1	29806294	0.84	0.84	0.65	0.64	0.71	0.52	0.49	0.81	34.99
10	s12004.1	30674002	0.46	0.37	0.40	0.71	0.74	0.33	0.73	0.45	25.29

10	OAR10_30746533.1	30700853	0.57	0.73	0.68	0.49	0.71	0.16	0.73	0.35	27.22
10	OAR10_40212146.1	39393584	0.62	0.28	0.52	0.27	0.53	0.42	0.49	0.71	22.89
10	OAR10_49074172.1	48300368	0.65	0.69	0.56	0.26	0.67	0.60	0.83	0.23	27.98
10	OAR10_54818443.1	53666062	0.74	0.74	0.70	0.45	0.65	0.69	0.46	0.58	31.99
10	OAR10_57152217.1	56010798	0.66	0.58	0.49	0.63	0.55	0.17	0.45	0.57	24.57
10	OAR10_61672235.1	60158728	0.35	0.35	0.55	0.53	0.84	0.81	0.45	0.33	25.79
10	s71442.1	82994149	0.71	0.80	0.44	0.25	0.72	0.50	0.12	0.66	25.19
10	s13236.1	84812819	0.46	0.50	0.48	0.47	0.50	0.46	0.46	0.46	22.58
10	OAR10_93289640.1	85627106	0.39	0.74	0.50	0.65	0.16	0.53	0.22	0.59	22.65
11	OAR11_11214325.1	11626457	0.87	0.87	0.55	0.33	0.71	0.80	0.67	0.22	31.92
11	s17310.1	19385272	0.88	0.78	0.86	0.51	0.28	0.56	0.91	0.75	36.00
11	OAR11_19800228_X.1	19397667	0.76	0.69	0.69	0.29	0.29	0.26	0.74	0.61	26.64
11	s49226.1	22736347	0.48	0.51	0.52	0.55	0.58	0.50	0.51	0.47	24.80
11	OAR11_23673049.1	22780752	0.70	0.70	0.56	0.56	0.71	0.43	0.67	0.61	30.93
11	s41017.1	22814442	0.71	0.71	0.55	0.60	0.72	0.41	0.69	0.63	31.59
11	OAR11_27654920.1	26421905	0.73	0.73	0.48	0.62	0.74	0.70	0.44	0.73	32.60
11	s10224.1	43937146	0.62	0.67	0.57	0.55	0.66	0.44	0.45	0.36	26.42
11	s50510.1	45436064	0.58	0.50	0.54	0.25	0.43	0.49	0.60	0.65	24.42
11	OAR11_52263542.1	49193942	0.60	0.55	0.53	0.42	0.70	0.43	0.62	0.58	27.17
12	OAR12_39493157.1	35320580	0.64	0.59	0.57	0.69	0.80	0.28	0.51	0.79	30.25
12	OAR12_43257167.1	38843578	0.71	0.51	0.73	0.62	0.79	0.71	0.63	0.88	35.84
12	OAR12_43331187.1	38923821	0.69	0.91	0.76	0.56	0.95	0.69	0.53	0.82	38.33
12	OAR12_43408733.1	39002907	0.71	0.65	0.60	0.38	0.73	0.46	0.40	0.88	29.85
12	OAR12_50496675.1	45600886	0.44	0.49	0.65	0.64	0.82	0.28	0.51	0.69	27.64
12	OAR12_71435396.1	64847102	0.57	0.16	0.69	0.48	0.29	0.47	0.76	0.66	25.01
12	OAR12_71458988.1	64870223	0.62	0.43	0.50	0.61	0.43	0.43	0.48	0.53	24.28

13	OAR13_51387503.1	48147260	0.72	0.53	0.48	0.46	0.77	0.46	0.86	0.80	31.72
13	OAR13_63161846.1	58023662	0.44	0.64	0.49	0.39	0.65	0.57	0.47	0.52	25.34
13	OAR13_81527927.1	75702438	0.68	0.75	0.16	0.37	0.57	0.33	0.23	0.77	22.50
13	s75919.1	78004271	0.60	0.66	0.51	0.44	0.69	0.41	0.66	0.46	27.18
13	s46185.1	78420224	0.56	0.65	0.80	0.64	0.20	0.52	0.68	0.55	28.94
14	s71975.1	16349731	0.58	0.71	0.46	0.41	0.36	0.71	0.33	0.58	25.21
14	OAR14_21510686_X.1	20924529	0.58	0.72	0.09	0.58	0.58	0.68	0.16	0.47	22.65
14	OAR14_37495397.1	36031629	0.66	0.57	0.42	0.47	0.64	0.63	0.41	0.48	25.96
14	OAR14_39834952.1	38250883	0.26	0.53	0.74	0.37	0.38	0.29	0.53	0.75	22.98
14	OAR14_52888836.1	49997609	0.67	0.64	0.44	0.20	0.68	0.50	0.42	0.41	23.80
15	OAR15_13425521.1	13417873	0.82	0.90	0.50	0.29	0.88	0.76	0.82	0.45	34.56
15	OAR15_32949553.1	31492057	0.25	0.53	0.48	0.54	0.70	0.59	0.44	0.83	26.54
15	s72653.1	31723078	0.39	0.47	0.29	0.67	0.52	0.46	0.46	0.75	23.77
15	OAR15_44332191.1	42207222	0.76	0.76	0.69	0.17	0.77	0.74	0.76	0.50	32.88
15	OAR15_58554489.1	53412021	0.67	0.47	0.55	0.56	0.31	0.45	0.40	0.52	23.82
15	OAR15_68933568.1	63386447	0.51	0.68	0.34	0.29	0.47	0.66	0.61	0.63	25.37
15	s28875.1	72707350	0.65	0.89	0.52	0.29	0.81	0.77	0.71	0.57	33.05
15	s52687.1	72747023	0.69	0.67	0.02	0.48	0.73	0.62	0.41	0.37	23.58
15	OAR15_87767502.1	79011094	0.54	0.66	0.58	0.56	0.65	0.50	0.69	0.20	27.14
16	OAR16_8234966.1	7731038	0.54	0.55	0.61	0.59	0.75	0.70	0.38	0.71	30.28
16	OAR16_26522451.1	24393554	0.38	0.55	0.73	0.45	0.55	0.90	0.69	0.71	31.69
16	OAR16_33317775.1	30655866	0.34	0.71	0.45	0.37	0.65	0.39	0.51	0.68	24.50
16	OAR16_33435828.1	30720540	0.54	0.73	0.50	0.38	0.65	0.09	0.56	0.60	24.08
16	OAR16_33478147.1	30847022	0.49	0.63	0.32	0.49	0.76	0.50	0.34	0.63	24.92
16	s51002.1	70900966	0.70	0.75	0.36	0.75	0.82	0.21	0.49	0.40	27.03
16	s37581.1	70922464	0.70	0.75	0.36	0.64	0.82	0.21	0.49	0.40	26.23

17	OAR17_1759664.1	1440961	0.44	0.58	0.53	0.55	0.59	0.16	0.55	0.61	23.81
17	OAR17_1818548.1	1509630	0.90	0.88	0.71	0.62	0.83	0.59	0.67	0.73	38.34
17	s36178.1	12319170	0.42	0.84	0.44	0.53	0.75	0.25	0.44	0.59	25.49
17	OAR17_13903096.1	12471246	0.42	0.69	0.55	0.33	0.65	0.49	0.28	0.80	25.65
17	OAR17_28390642.1	25862600	0.43	0.46	0.64	0.15	0.63	0.38	0.54	0.53	22.48
17	s09129.1	53470559	0.67	0.56	0.33	0.39	0.83	0.80	0.63	0.76	31.04
17	s49475.1	53499969	0.71	0.65	0.36	0.42	0.85	0.82	0.65	0.78	33.00
17	OAR17_58220718.1	53511490	0.71	0.65	0.33	0.42	0.85	0.36	0.58	0.78	28.51
17	s05080.1	54645722	0.42	0.56	0.55	0.32	0.58	0.52	0.30	0.71	23.76
18	s31152.1	19342316	0.61	0.55	0.50	0.17	0.97	0.58	0.71	0.52	28.51
18	s62564.1	19399095	0.57	0.78	0.32	0.12	0.79	0.60	0.54	0.38	24.62
18	s12590.1	20040105	0.84	0.65	0.59	0.49	0.82	0.62	0.75	0.52	33.64
18	OAR18_47726031.1	44805751	0.32	0.58	0.78	0.50	0.77	0.28	0.27	0.68	25.40
18	OAR18_47790447_X.1	44858471	0.32	0.58	0.78	0.50	0.77	0.42	0.27	0.68	26.50
18	s29776.1	68278290	0.50	0.61	0.75	0.74	0.86	0.45	0.57	0.83	33.78
19	OAR19_17468739_X.1	16713971	0.55	0.40	0.40	0.66	0.36	0.61	0.63	0.62	25.79
19	OAR19_32257190.1	30608973	0.51	0.18	0.60	0.44	0.54	0.40	0.68	0.80	25.13
19	OAR19_33593848.1	31915771	0.63	0.27	0.75	0.33	0.14	0.48	0.78	0.67	24.90
19	s04445.1	53852012	0.49	0.64	0.40	0.32	0.79	0.26	0.66	0.71	25.68
19	s21513.1	55690132	0.76	0.65	0.38	0.59	0.81	0.77	0.76	0.42	32.46
20	s02472.1	15603841	0.62	0.71	0.72	0.29	0.65	0.54	0.35	0.40	26.36
20	OAR20_37437726.1	34168829	0.74	0.86	0.73	0.75	0.62	0.43	0.69	0.45	33.58
20	s39515.1	34256995	0.54	0.64	0.70	0.64	0.42	0.32	0.62	0.22	25.06
21	s37379.1	30761604	0.44	0.65	0.52	0.42	0.49	0.32	0.71	0.66	25.54
21	OAR21_50378541.1	45368906	0.62	0.74	0.35	0.58	0.75	0.25	0.51	0.64	26.77
22	OAR22_20201826.1	16421983	0.69	0.77	0.38	0.61	0.80	0.35	0.07	0.49	24.72

22	OAR22_48326768.1	42969386	0.48	0.57	0.45	0.47	0.58	0.57	0.45	0.59	25.17
22	s34755.1	43123707	0.51	0.69	0.68	0.80	0.45	0.49	0.47	0.50	28.64
22	s47761.1	49298680	0.55	0.52	0.48	0.57	-0.01	0.66	0.49	0.57	23.20
23	OAR23_27062541.1	25919106	0.72	0.70	0.41	0.27	0.71	0.70	0.72	0.15	27.00
23	OAR23_27112379.1	25972928	0.86	0.93	0.82	0.57	0.90	0.90	0.80	0.95	44.46
23	OAR23_34033124.1	32354664	0.39	0.55	0.49	0.55	0.62	0.43	0.50	0.40	23.55
23	OAR23_44089565.1	41621459	0.60	0.54	0.73	0.54	0.71	0.40	0.19	0.62	26.42
23	OAR23_61577887.1	57787391	0.65	0.67	0.60	0.62	0.06	0.35	0.67	0.45	24.88
23	OAR23_63417651.1	59418377	0.70	0.78	0.60	0.34	0.78	0.39	0.73	0.75	31.74
24	s59452.1	9721533	0.73	0.86	0.44	0.72	0.52	0.28	0.27	0.73	27.83
24	s00643.1	24846522	0.81	0.87	0.24	0.63	0.56	0.13	0.20	0.76	24.72
24	OAR24_42942982.1	39726323	0.57	0.57	0.28	0.82	0.26	0.45	0.71	0.74	26.84
25	s42703.1	17652415	0.78	0.90	0.66	0.72	0.75	0.56	0.32	0.84	35.20
25	OAR25_20106030.1	19438048	0.70	0.59	0.56	0.29	0.81	0.49	0.29	0.69	26.95
25	s72346.1	42689823	0.80	0.54	0.47	0.23	0.50	0.45	0.63	0.23	23.05

Notes: Significant SNPs located within merged genomic regions are colored "red".

Table S4. Significant SNPs identified in Changthangi sheep

CHR	SNP	Position	Fst between Changthangi and								Di
			BanglBGE	BanglGarole	Deccani	Garut	IndianGarole	Kage	Lampuchhre	Sumatra	
1	s41913.1	2870378	0.55	0.44	0.54	0.59	0.62	0.49	0.14	0.60	25.31
1	OAR1_20035449.1	19970769	0.64	0.52	0.42	0.66	0.48	0.63	0.48	0.39	27.29
1	OAR1_72984979.1	68457284	0.56	0.60	0.49	0.44	0.57	0.05	0.56	0.48	23.07
1	OAR1_81010882.1	75815312	0.77	0.55	0.24	0.58	0.41	0.28	0.28	0.54	22.27
1	OAR1_107239370.1	99931717	0.50	0.65	0.45	0.40	0.63	0.37	0.65	0.58	27.08
1	OAR1_107504871.1	100185774	0.64	0.64	0.62	0.61	0.65	0.60	0.64	0.46	32.27
1	OAR1_145497863.1	134502219	0.56	0.34	0.21	0.65	0.58	0.65	0.33	0.25	22.08
1	OAR1_156411137.1	144907948	0.59	0.55	0.52	0.43	0.59	0.19	0.38	0.68	24.66
1	s13827.1	172607565	0.58	0.47	0.50	0.13	0.70	-0.02	0.37	0.77	21.13
1	OAR1_199252158.1	184627258	0.53	0.59	0.42	0.30	0.70	0.68	0.44	0.71	28.24
1	OAR1_205303256.1	190370051	0.43	0.61	0.63	0.13	0.51	0.48	0.66	0.44	24.86
1	OAR1_223784851.1	207248370	0.60	0.72	0.49	0.48	0.73	0.24	0.09	0.62	24.82
1	s32029.1	210073609	0.69	0.79	0.22	0.52	0.75	0.65	0.09	0.19	24.49
1	OAR1_237504699.1	220288059	0.50	0.64	0.26	0.57	0.55	0.54	0.58	0.45	25.88
1	s68196.1	247826668	0.51	0.70	0.09	0.21	0.80	0.46	0.58	0.39	22.69
1	OAR1_268122939.1	248275678	0.94	0.82	0.69	0.35	0.65	0.36	0.60	0.57	32.97
1	OAR1_273868392.1	253325565	0.58	0.51	0.48	0.54	0.31	0.52	0.37	0.11	21.29
2	OAR2_7954574.1	8942592	0.52	0.65	0.37	-0.01	0.66	0.48	0.42	0.64	23.02
2	OAR2_16538077.1	16975392	0.14	0.66	0.62	0.37	0.29	0.28	0.54	0.63	21.90
2	OAR2_31888274.1	30919873	0.71	0.77	0.54	0.16	0.32	0.68	0.49	0.63	28.04
2	OAR2_32180338.1	31018254	0.41	0.57	0.51	0.23	0.28	0.50	0.52	0.72	23.58
2	OAR2_39605749.1	38050036	0.46	0.55	0.38	0.36	0.47	0.41	0.62	0.32	22.14
2	OAR2_60859576.1	56556228	0.61	0.79	0.54	0.30	0.54	0.21	0.17	0.57	23.20

2	OAR2_64231337.1	59762935	0.32	0.25	0.15	0.60	0.46	0.30	0.82	0.65	21.46
2	OAR2_86948508.1	81785728	0.69	0.82	0.58	0.67	0.42	0.42	0.29	0.68	29.85
2	OAR2_87089443.1	81887124	0.32	0.74	0.38	0.51	0.49	0.39	0.62	0.62	25.73
2	OAR2_87543957.1	82346323	0.44	0.34	0.55	0.46	0.46	0.09	0.58	0.61	21.76
2	OAR2_88927720.1	83644533	0.40	0.66	0.57	0.68	0.51	0.73	0.55	0.39	29.50
2	OAR2_107945014.1	100364649	0.71	0.74	0.33	0.64	0.51	0.41	0.22	0.76	27.50
2	s33638.1	129791658	0.62	0.62	0.23	0.39	0.56	0.17	0.54	0.61	22.82
2	OAR2_143916009.1	135304995	0.39	0.50	0.47	0.51	0.55	0.40	0.29	0.53	22.48
2	OAR2_171359957.1	161748310	0.61	0.72	0.18	0.39	0.81	0.67	0.50	0.51	27.99
2	OAR2_174148415.1	164516447	0.58	0.63	0.35	0.46	0.70	0.35	0.43	0.31	23.68
2	OAR2_174236715.1	164594319	0.60	0.56	0.34	0.57	0.50	0.55	0.45	0.44	25.41
2	OAR2_219736519.1	207512168	0.49	0.42	0.68	0.28	0.70	0.13	0.71	0.15	22.18
2	s33598.1	217834549	0.75	0.75	0.33	0.40	0.66	0.63	0.39	0.19	26.12
2	s37310.1	232414241	0.61	0.44	0.47	0.10	0.62	0.57	0.54	0.24	22.64
2	s13779.1	232485668	0.85	0.56	0.71	0.39	0.88	0.85	0.16	0.63	33.66
2	s09024.1	234609188	0.50	0.39	0.36	0.44	0.67	0.11	0.66	0.45	21.77
2	s41966.1	237813990	0.42	0.74	0.72	0.05	0.65	0.56	0.58	0.69	28.75
2	OAR2_251166495.1	237822440	0.42	0.74	0.72	0.05	0.65	0.56	0.58	0.69	28.75
2	OAR2_259153647.1	245206162	0.74	0.66	0.32	0.74	0.73	0.41	0.43	0.11	26.25
3	OAR3_42867624.1	40124759	0.73	0.57	0.30	0.25	0.70	0.51	0.41	0.03	21.54
3	OAR3_44467884.1	41551185	0.58	0.32	0.20	0.55	0.24	0.53	0.51	0.61	21.71
3	s25321.1	62015360	0.68	0.68	0.62	0.61	0.69	0.64	0.65	0.64	34.87
3	s68356.1	62044832	0.64	0.64	0.53	0.49	0.61	0.60	0.60	0.59	30.83
3	OAR3_66317995.1	62704565	0.78	0.40	0.25	0.58	0.42	0.35	0.49	0.45	23.09
3	OAR3_67654203.1	64054051	0.44	0.77	0.35	0.64	0.25	0.39	0.10	0.58	21.56
3	OAR3_77361732.1	73231766	0.76	0.31	0.27	0.55	0.20	0.18	0.64	0.68	21.95

3	s09462.1	104064729	0.49	0.41	0.09	0.24	0.64	0.47	0.68	0.52	21.26
3	OAR3_115417890_X.1	108409583	0.57	0.39	0.55	0.54	0.58	0.19	0.30	0.53	22.63
3	OAR3_115425020_X.1	108415853	0.55	0.37	0.53	0.52	0.56	0.21	0.27	0.50	21.58
3	OAR3_115443384.1	108434471	0.55	0.58	0.78	0.48	0.85	0.30	0.40	0.76	30.82
3	OAR3_135754645.1	127278076	0.53	0.38	0.46	0.41	0.54	0.49	0.53	0.11	21.45
3	OAR3_141010154.1	131915865	0.50	0.64	0.72	0.49	0.87	0.23	0.34	0.55	27.90
3	OAR3_150785833.1	140970538	0.69	0.38	0.66	0.68	0.36	0.41	0.77	0.82	31.58
3	OAR3_163942175.1	153280516	0.53	0.65	0.38	0.54	0.33	0.49	0.58	0.57	25.95
3	OAR3_165009241.1	154213690	0.16	0.13	0.88	0.79	0.06	0.43	0.94	0.69	26.88
3	OAR3_166530239_X.1	155660948	0.50	0.36	0.67	0.52	0.67	0.11	0.11	0.72	22.70
3	OAR3_171973029.1	160758526	0.76	0.64	0.20	0.30	0.63	0.57	0.08	0.57	23.05
3	OAR3_174102523.1	162819252	0.55	0.55	0.53	0.48	0.56	0.51	0.55	0.55	27.83
3	s35132.1	163307018	0.70	0.79	0.47	0.65	0.86	0.74	0.25	0.36	31.67
3	s37564.1	165413630	0.69	0.45	0.49	0.70	0.25	0.45	0.07	0.46	22.29
3	s52576.1	165785278	0.39	0.55	0.76	0.15	0.79	0.56	0.58	0.25	26.14
3	s05280.1	178423049	0.67	0.44	0.38	0.18	0.07	0.65	0.64	0.35	21.25
3	s27086.1	178502319	0.70	0.50	0.41	0.02	0.30	0.55	0.39	0.55	21.20
3	OAR3_191756128.1	178517886	0.56	0.56	0.57	0.25	0.43	0.72	0.66	0.14	25.31
3	s60816.1	178743725	0.72	0.74	0.41	0.74	0.46	0.15	0.50	0.76	28.71
3	OAR3_194730311.1	180817708	0.41	0.47	0.42	0.66	0.26	0.02	0.66	0.62	21.55
3	OAR3_194751219.1	180839858	0.55	0.63	0.53	0.39	0.38	0.15	0.66	0.68	25.14
3	s70839.1	186368226	0.51	0.51	0.47	0.42	0.63	0.49	0.51	0.54	25.94
3	OAR3_202159984.1	187895177	0.62	0.00	0.57	0.50	0.39	0.42	0.62	0.39	22.08
4	s53485.1	9377742	0.34	0.43	0.56	0.40	0.40	0.17	0.77	0.48	22.15
4	OAR26_43350073.1	12666865	0.60	0.52	0.53	0.36	0.61	0.51	0.60	0.55	27.59
4	OAR4_14572146.1	14355529	0.73	0.66	0.50	0.57	0.59	0.47	0.77	0.82	33.88

4	OAR4_24779248.1	23648876	0.51	0.62	0.33	0.51	0.63	0.49	0.08	0.28	21.00
4	OAR4_28903984.1	27527718	0.68	0.57	0.23	0.23	0.76	0.31	0.45	0.55	23.14
4	s32219.1	48304254	0.44	0.25	0.38	0.85	0.22	0.46	0.82	0.91	27.93
4	OAR4_51441757.1	48627750	0.47	0.47	0.39	0.30	0.48	0.42	0.47	0.46	21.14
4	OAR4_51489408.1	48669962	0.69	0.59	0.67	0.59	0.73	0.69	0.72	0.72	36.44
4	OAR4_71992063.1	68012278	0.55	0.64	0.00	0.36	0.60	0.45	0.40	0.57	21.31
4	OAR4_80559387.1	75968047	0.40	0.61	0.44	0.58	0.78	0.41	0.79	0.87	31.74
4	OAR4_80605717.1	76007878	0.38	0.59	0.32	0.20	0.70	0.57	0.71	0.44	24.41
4	s11336.1	87572495	0.55	0.55	0.53	0.52	0.56	0.37	0.55	0.55	26.86
4	OAR4_93067608.1	87649810	0.64	0.60	0.30	0.45	0.59	0.17	0.48	0.56	23.30
5	OAR5_12579856.1	11026335	0.70	0.48	0.61	0.64	0.53	0.24	0.05	0.16	21.08
5	s29567.1	16203101	0.50	0.80	0.82	0.30	0.78	0.31	0.75	0.14	28.83
5	OAR5_29893442.1	26851727	0.64	0.69	0.26	0.49	0.63	0.39	0.30	0.10	21.24
5	OAR5_42039615.1	38259222	0.61	0.51	0.22	0.65	0.31	0.47	0.18	0.57	21.60
5	s46892.1	40487686	0.41	0.49	0.42	0.46	0.50	0.30	0.49	0.48	21.84
5	s72060.1	40713566	0.61	0.79	0.70	0.09	0.65	0.35	0.39	0.66	27.30
5	s23065.1	41357859	0.54	0.67	0.33	0.11	0.47	0.55	0.67	0.53	24.34
5	OAR5_53338330.1	49080645	0.39	0.64	0.38	0.46	0.69	0.28	0.58	0.61	25.22
5	OAR5_56001782.1	51587928	0.62	0.52	0.57	0.61	0.48	0.35	0.25	0.49	24.71
5	OAR5_56257273.1	51843084	0.75	0.56	0.62	0.22	0.61	0.60	0.26	0.35	25.43
5	OAR5_88516298.1	80558908	0.39	0.72	0.30	0.70	0.55	0.20	0.21	0.56	21.95
5	OAR5_104570871.1	96006551	0.59	0.77	0.29	0.85	0.44	0.32	0.45	0.63	27.58
6	OAR6_47518072.1	42568830	0.03	0.29	0.51	0.77	0.49	0.35	0.39	0.60	21.03
6	s55289.1	46602091	0.69	0.61	0.48	0.31	0.61	0.27	0.31	0.37	22.53
6	OAR6_56272175.1	51016603	0.36	0.55	0.35	0.44	0.89	0.43	0.23	0.30	21.72
6	OAR6_59327164.1	53995243	0.54	0.44	0.63	0.25	0.59	0.09	0.56	0.48	22.12

6	s21552.1	94639943	0.57	0.65	0.80	0.07	0.82	0.84	0.29	0.61	30.93
6	OAR6_121352193.1	106834499	0.47	0.73	0.26	0.33	0.54	0.32	0.64	0.51	23.47
6	s32537.1	115101978	0.47	0.64	0.26	0.41	0.64	0.53	0.65	0.63	26.84
7	OAR7_4883405.1	5081808	0.58	0.58	0.51	0.42	0.59	0.54	0.54	0.57	27.90
7	s24872.1	15988141	0.37	0.35	0.60	0.62	0.73	0.63	0.23	0.60	26.47
7	s17550.1	42463209	0.36	0.61	0.34	0.50	0.31	0.60	0.12	0.61	21.25
7	OAR7_47161420.1	42551974	0.31	0.68	0.42	0.58	0.34	0.71	0.36	0.52	25.13
7	OAR7_52060307.1	47264055	0.81	0.70	0.36	0.17	0.60	0.71	0.38	0.50	27.20
7	OAR7_63627909.1	57692469	0.64	0.55	0.71	0.40	0.52	0.81	0.69	0.76	34.34
7	OAR7_63692612.1	57764872	0.69	0.71	0.78	0.52	0.60	0.83	0.61	0.73	37.23
7	OAR7_63745942.1	57816492	0.79	0.77	0.88	0.61	0.90	0.84	0.89	0.89	45.69
7	OAR7_63814443.1	57875478	0.94	0.94	0.93	0.65	0.94	0.90	0.89	0.88	49.40
7	OAR7_63848145.1	57915106	0.91	0.91	0.91	0.90	0.92	0.90	0.91	0.91	51.10
7	OAR7_83320136.1	76232943	0.42	0.63	0.43	0.25	0.71	0.35	0.48	0.56	23.70
7	OAR7_87066180.1	79817839	0.47	0.67	0.35	-0.02	0.69	0.70	0.19	0.46	21.51
7	OAR7_87079540.1	79831939	0.56	0.53	0.44	0.38	0.40	0.42	0.40	0.59	23.43
7	OAR7_89553402.1	82197428	0.42	0.34	0.57	0.28	0.68	0.45	0.44	0.79	25.16
7	OAR7_97159644.1	89286934	0.53	0.57	0.39	0.20	0.39	0.38	0.43	0.63	21.75
7	OAR7_97378846.1	89519883	0.55	0.55	0.58	0.17	0.66	0.21	0.68	0.06	21.34
7	OAR7_101493967.1	93353104	0.55	0.91	0.38	0.21	0.51	0.42	-0.01	0.54	21.57
8	s72510.1	10725738	0.33	0.47	0.54	0.26	0.44	0.48	0.57	0.53	22.85
8	OAR8_12125386.1	10820850	0.31	0.59	0.56	0.44	0.64	0.58	0.53	0.42	26.15
8	s34333.1	47947284	0.71	0.36	0.14	0.28	0.75	0.51	0.53	0.65	24.38
8	OAR8_55339073.1	51474008	0.42	0.49	0.13	0.32	0.67	0.57	0.59	0.41	21.99
8	OAR8_67469564.1	62674808	0.31	0.41	0.47	0.50	0.59	0.24	0.61	0.50	22.46
9	s35553.1	18169154	0.47	0.62	0.55	0.55	0.29	0.28	0.41	0.54	23.24

9	OAR9_45952110.1	43803853	0.03	0.50	0.70	0.42	0.60	0.53	0.75	0.57	26.56
9	OAR9_59326545.1	56572469	0.45	0.45	0.60	0.03	0.67	0.65	0.26	0.29	21.17
9	OAR9_64769748.1	61628833	0.69	0.24	0.66	0.52	0.42	0.56	0.78	0.28	27.38
9	OAR9_64800745_X.1	61661127	0.11	0.36	0.37	0.44	0.57	0.52	0.72	0.34	21.01
9	OAR9_66491264.1	63052885	0.31	0.46	0.41	0.51	0.60	0.45	0.16	0.65	21.83
9	OAR9_79964970.1	75412590	0.62	0.62	0.44	0.59	0.63	0.53	0.16	0.34	24.80
9	OAR9_81909760.1	77297358	0.46	0.79	0.44	0.82	0.58	0.40	0.79	0.86	33.76
9	OAR9_82428531.1	77756891	0.59	0.87	0.46	0.44	0.48	0.50	0.83	0.91	33.58
10	OAR10_29092520.1	29054709	0.60	0.50	0.41	0.21	0.53	0.76	0.74	0.46	27.29
10	OAR10_29469450.1	29436435	0.66	0.66	0.64	0.36	0.63	0.58	0.59	0.62	31.34
10	OAR10_29511510.1	29476678	0.68	0.68	0.66	0.65	0.69	0.64	0.68	0.64	35.75
10	OAR10_29538398.1	29502667	0.57	0.67	-0.02	0.56	0.19	0.38	0.60	0.66	21.85
10	OAR10_53872811.1	52811394	0.55	0.24	0.54	0.73	0.65	0.28	0.29	0.25	21.87
10	OAR10_57152217.1	56010798	0.82	0.74	0.65	0.80	0.70	0.34	0.61	0.73	36.04
11	OAR11_19632538.1	19223203	0.72	0.60	0.46	0.52	0.73	0.45	0.53	0.56	29.65
11	s17310.1	19385272	0.74	0.61	0.71	0.27	0.09	0.31	0.79	0.57	26.80
11	OAR11_19800228_X.1	19397667	0.73	0.64	0.64	0.21	0.21	0.19	0.70	0.55	24.63
11	OAR11_23673049.1	22780752	0.62	0.62	0.44	0.43	0.63	0.28	0.58	0.50	25.95
11	s41017.1	22814442	0.62	0.62	0.40	0.47	0.63	0.25	0.58	0.50	25.60
11	OAR11_43313971.1	40826416	0.57	0.43	0.39	0.45	0.07	0.47	0.60	0.56	22.17
11	s46554.1	49142365	0.44	0.44	0.51	0.22	0.59	0.21	0.54	0.71	22.63
11	OAR11_52263542.1	49193942	0.52	0.48	0.45	0.34	0.63	0.34	0.55	0.50	23.84
12	OAR12_29127884.1	25645126	0.53	0.35	0.13	0.51	0.58	0.72	0.39	0.30	21.48
12	OAR12_38753500.1	34604355	0.71	0.58	0.62	0.52	0.68	0.72	0.71	0.18	31.57
12	OAR12_43257167.1	38843578	0.50	0.26	0.51	0.37	0.60	0.47	0.39	0.73	24.02
12	OAR12_43331187.1	38923821	0.53	0.82	0.60	0.35	0.87	0.49	0.35	0.69	30.68

12	OAR12_43408733.1	39002907	0.61	0.53	0.47	0.24	0.63	0.31	0.26	0.81	24.14
12	OAR12_50496675.1	45600886	0.43	0.48	0.66	0.64	0.85	0.28	0.51	0.71	29.55
12	s21761.1	47186720	0.26	0.71	0.26	0.19	0.69	0.26	0.71	0.61	22.44
13	OAR13_14471555.1	14235819	0.80	0.51	0.28	0.44	0.81	0.52	-0.02	0.11	21.02
13	s14792.1	32586633	0.72	0.47	0.42	0.59	0.41	0.30	0.45	0.08	21.33
13	s60097.1	52962246	0.51	0.94	0.28	0.54	0.64	0.28	0.32	0.45	24.52
13	OAR13_67400889.1	62269037	0.64	0.44	0.65	0.40	0.47	0.16	0.21	0.43	20.98
13	OAR13_81921734_X.1	76099369	0.55	0.55	0.53	0.52	0.56	0.21	0.55	0.55	25.48
13	s75919.1	78004271	0.63	0.69	0.53	0.44	0.73	0.41	0.69	0.46	29.81
14	OAR14_4087361.1	4147946	0.50	0.58	0.51	0.27	0.45	0.40	0.54	0.57	24.03
14	OAR14_36559626.1	35119271	0.44	0.70	0.15	0.53	0.52	0.74	0.13	0.37	22.06
14	OAR14_37495397.1	36031629	0.68	0.58	0.40	0.46	0.66	0.64	0.39	0.47	27.72
14	OAR14_51700380_X.1	49082845	0.36	0.50	0.02	0.72	0.52	0.71	0.50	0.17	21.23
14	OAR14_52888836.1	49997609	0.64	0.60	0.35	0.12	0.65	0.42	0.34	0.33	21.00
14	s40432.1	56451397	0.51	0.42	0.36	0.70	0.82	0.25	0.47	0.63	26.11
15	OAR15_44198795.1	42070930	0.47	0.53	0.19	0.39	0.62	0.42	0.44	0.66	22.69
15	OAR15_44332191.1	42207222	0.59	0.59	0.49	-0.01	0.60	0.55	0.59	0.25	23.01
15	OAR15_68933568.1	63386447	0.41	0.61	0.23	0.18	0.37	0.57	0.53	0.54	20.99
15	OAR15_87767502.1	79011094	0.41	0.56	0.45	0.43	0.55	0.36	0.59	0.08	21.18
16	OAR16_33435828.1	30720540	0.52	0.73	0.48	0.37	0.63	0.10	0.54	0.58	24.72
16	OAR16_37226202.1	34279856	0.69	0.74	0.66	0.44	0.68	0.35	0.62	0.68	32.04
16	s44596.1	34315060	0.64	0.59	0.63	-0.02	0.64	0.35	0.62	0.61	25.95
16	s15191.1	39007570	0.66	0.53	0.67	0.48	0.67	0.57	0.66	0.56	31.87
16	OAR16_48105833.1	44145304	0.56	0.46	0.16	0.49	0.48	0.26	0.56	0.63	21.85
16	OAR16_69511009.1	63750355	0.60	0.83	0.66	0.54	0.92	0.66	0.58	0.29	33.82
17	OAR17_3467332.1	3034628	0.36	0.31	0.74	0.49	0.30	0.25	0.43	0.60	21.82

17	OAR17_11520692.1	10425201	0.45	0.60	0.41	0.29	0.57	0.56	0.49	0.48	24.25
17	s36178.1	12319170	0.34	0.82	0.36	0.46	0.70	0.18	0.37	0.53	22.88
17	OAR17_23888723.1	21434752	0.71	0.54	0.59	0.22	0.43	0.60	0.70	0.67	29.33
17	OAR17_23905156.1	21454166	0.69	0.55	0.44	0.13	0.21	0.66	0.53	0.68	24.92
17	s36776.1	54064665	0.66	0.29	0.53	0.43	0.54	0.51	0.10	0.73	23.98
18	s31152.1	19342316	0.51	0.45	0.39	0.07	0.94	0.45	0.63	0.41	23.92
18	s62564.1	19399095	0.55	0.80	0.29	0.10	0.81	0.59	0.53	0.35	25.35
18	s12590.1	20040105	0.77	0.52	0.45	0.33	0.74	0.47	0.64	0.37	27.69
18	OAR18_52089434.1	48867859	0.33	0.38	0.44	0.49	0.23	0.69	0.80	0.03	21.41
18	OAR18_59577534.1	55762626	0.59	0.56	0.54	0.27	0.13	0.25	0.57	0.63	22.06
18	s48176.1	67289143	0.73	0.78	0.74	0.12	0.62	0.53	0.73	0.45	31.17
18	s29776.1	68278290	0.25	0.37	0.54	0.52	0.70	0.17	0.33	0.66	21.55
19	OAR19_4318651.1	4077028	0.67	0.69	0.71	0.68	0.53	0.65	0.27	0.61	31.99
19	s25034.1	48259482	0.28	0.33	0.39	0.47	0.33	0.48	0.74	0.43	21.36
19	s04445.1	53852012	0.42	0.58	0.32	0.24	0.77	0.18	0.61	0.66	23.06
19	s21513.1	55690132	0.59	0.44	0.16	0.37	0.66	0.58	0.59	0.19	21.88
19	OAR19_61334312.1	57708741	0.60	0.60	0.57	0.48	0.61	0.42	0.60	0.17	26.04
20	OAR20_9024546_X.1	8893097	0.55	0.55	0.25	0.39	0.45	0.51	0.47	0.43	22.26
20	OAR20_37437726.1	34168829	0.69	0.84	0.68	0.70	0.56	0.35	0.64	0.37	32.03
20	s39515.1	34256995	0.47	0.59	0.67	0.59	0.34	0.23	0.56	0.14	22.65
20	OAR20_48476815.1	44558460	0.70	0.57	0.21	0.53	0.68	0.50	0.51	0.56	26.99
21	OAR21_12460436.1	10911345	0.74	0.69	0.48	0.52	0.24	0.58	0.58	0.65	29.34
21	s37379.1	30761604	0.39	0.61	0.48	0.37	0.45	0.28	0.69	0.63	24.47
21	s63819.1	43818339	0.45	0.16	0.47	0.65	0.27	0.74	0.50	0.65	25.00
21	s65606.1	46568117	0.26	0.39	0.55	0.54	0.39	0.54	0.60	0.65	25.03
22	OAR22_33483867.1	29116134	0.57	0.50	0.58	0.53	0.52	0.47	0.34	0.63	26.64

23	s69818.1	3075709	0.64	0.61	0.26	0.40	0.63	0.43	0.58	0.60	26.23
23	OAR23_27112379.1	25972928	0.64	0.76	0.57	0.24	0.71	0.69	0.55	0.78	32.81
23	OAR23_34033124.1	32354664	0.36	0.56	0.49	0.55	0.63	0.41	0.49	0.39	24.43
23	s31567.1	51126740	0.74	0.52	0.75	0.81	0.70	0.59	0.80	0.30	35.38
23	OAR23_66632347.1	62242039	0.77	0.80	0.29	0.26	0.59	0.62	0.52	0.58	28.51
24	s27883.1	9627147	0.55	0.63	0.44	0.61	0.43	0.14	0.40	0.44	22.57
24	s45778.1	9658771	0.64	0.72	0.49	0.53	0.53	0.25	0.29	0.53	25.02
24	s59452.1	9721533	0.77	0.91	0.48	0.77	0.55	0.34	0.32	0.77	31.96
24	s69582.1	37753985	0.69	0.34	0.46	0.59	0.67	0.17	0.56	0.65	26.03
25	s68395.1	31303297	0.47	0.63	0.47	0.18	0.49	0.49	0.31	0.57	22.45
26	OAR26_17518698.1	14644438	0.74	0.31	0.26	0.46	0.68	0.62	0.47	0.24	23.70
26	OAR26_20367221.1	17232588	0.45	0.45	0.43	0.42	0.46	0.41	0.45	0.44	21.61
26	OAR26_34360075.1	30036364	0.51	0.66	0.33	0.49	0.58	0.48	0.39	0.36	23.70
26	s22112.1	42948826	0.63	0.39	0.44	0.39	0.73	0.31	0.55	0.57	25.35

Notes: Significant SNPs located within merged genomic regions are colored “red”.

Table S5. Significant SNPs identified in Baruwal sheep

CHR	SNP	Position	Fst between Baruwal and								Di
			BanglBGE	BanglGarole	Deccani	Garut	IndianGarole	Kage	Lampuchhre	Sumatra	
1	OAR1_9822302.1	10103593	0.70	0.77	0.45	0.74	0.46	0.47	0.70	0.67	22.54
1	OAR1_16786326.1	16801673	0.85	0.77	0.56	0.73	0.74	0.57	0.44	0.54	23.92
1	OAR1_34041305.1	33308065	0.69	0.82	0.89	0.70	0.70	0.62	0.32	0.84	26.37
1	OAR1_67773451.1	64101392	0.93	0.96	0.69	0.52	0.94	0.72	0.63	0.77	29.66
1	OAR1_69309059.1	65485724	0.52	0.43	0.53	0.68	0.59	0.89	0.68	0.79	23.73
1	OAR1_177013374.1	164069855	0.79	0.81	0.74	0.55	0.92	0.87	0.69	0.73	29.43
1	OAR1_181579456.1	168364994	0.74	0.77	0.47	0.71	0.65	0.18	0.72	0.85	23.06
1	OAR1_189179554.1	175482796	0.66	0.68	0.75	0.93	0.63	0.78	0.93	0.27	26.80
1	OAR1_191964562.1	178097316	0.29	0.87	0.70	0.74	0.45	0.81	0.41	0.86	23.63
1	s15220.1	182827903	0.69	0.67	0.85	0.61	0.66	0.52	0.72	0.44	24.03
1	OAR1_207317735.1	192074278	0.77	0.77	0.72	0.83	0.15	0.25	0.77	0.71	22.67
1	OAR1_217420572.1	201471554	0.77	0.61	0.75	0.30	0.73	0.48	0.50	0.79	22.55
1	OAR1_219772503.1	203499939	0.77	0.79	0.47	0.53	0.92	0.84	0.74	0.79	27.78
1	OAR1_261453067.1	241803266	0.92	0.79	0.64	0.78	0.96	0.68	0.51	0.76	28.84
1	s18320.1	243069237	0.94	0.98	0.73	0.93	0.98	0.00	0.54	0.83	27.80
1	OAR1_269956045.1	249966956	0.82	0.68	0.85	0.45	0.68	0.84	0.84	0.74	28.44
1	OAR1_270547664.1	250456371	0.54	0.64	0.67	0.43	0.81	0.54	0.82	0.79	24.19
1	OAR1_275446990.1	254866909	0.55	0.74	0.76	0.46	0.62	0.71	0.89	0.60	25.09
2	s63023.1	2991783	0.67	0.43	0.57	0.85	0.78	0.52	0.50	0.66	22.61
2	s58940.1	9701540	0.38	0.66	0.77	0.73	0.40	0.78	0.88	0.90	26.05
2	OAR2_52155350.1	48731082	0.69	0.89	0.40	0.76	0.66	0.56	0.69	0.68	24.70
2	OAR2_52187355.1	48762835	0.69	0.82	0.59	0.55	0.70	0.56	0.43	0.76	23.46
2	s31618.1	52177597	0.79	0.92	0.38	0.98	0.74	0.68	0.47	0.80	27.01

2	OAR2_55818455.1	52218919	0.67	0.82	0.31	0.88	0.71	0.55	0.33	0.79	22.89
2	OAR2_105762019.1	98330510	0.80	0.83	0.60	0.81	0.60	0.34	0.72	0.74	25.33
2	OAR2_107787796.1	100199475	0.80	0.82	0.75	0.74	0.65	0.47	0.41	0.96	26.28
2	OAR2_107945014.1	100364649	0.82	0.84	0.48	0.77	0.63	0.57	0.36	0.86	24.65
2	OAR2_132501035.1	124290905	0.87	0.85	0.83	0.86	0.85	0.41	0.69	0.55	28.16
2	s35762.1	139846639	0.52	0.62	0.71	0.61	0.73	0.28	0.82	0.73	22.83
2	s31000.1	140158515	0.68	0.94	0.44	0.30	0.84	0.44	0.77	0.54	22.38
2	OAR2_149959350.1	141063223	0.80	0.75	0.75	0.51	0.85	0.59	0.61	0.67	26.05
2	s07522.1	145693768	0.50	0.72	0.66	0.46	0.79	0.75	0.75	0.72	24.96
2	OAR2_154742668_X.1	145745763	0.64	0.79	0.64	0.78	0.70	0.70	0.38	0.96	26.25
2	OAR2_155715202.1	146734798	0.49	0.68	0.69	0.55	0.76	0.71	0.92	0.98	27.33
2	OAR2_155734672.1	146754173	0.51	0.60	0.74	0.55	0.42	0.71	0.94	0.98	25.57
2	s05571.1	182923305	0.50	0.71	0.65	0.75	0.39	0.70	0.45	0.76	22.39
2	OAR2_199892219.1	188490584	0.70	0.77	0.58	0.74	0.66	0.32	0.67	0.67	23.33
2	OAR2_213093422.1	201345538	0.96	1.00	0.93	0.52	0.82	0.76	1.00	0.36	31.08
2	OAR2_246880633.1	233775867	0.58	0.69	0.66	0.68	0.68	0.48	0.64	0.71	23.46
2	OAR2_252756874.1	239288339	0.70	0.70	0.61	0.88	0.62	0.47	0.87	0.29	23.74
3	s72862.1	920144	0.85	0.74	0.64	0.73	0.94	0.59	0.39	0.65	25.94
3	s24198.1	13936611	0.73	0.84	0.48	0.49	0.51	0.41	0.93	0.69	23.25
3	OAR3_17669643.1	16189589	0.78	0.53	0.52	0.52	0.68	0.59	0.51	0.80	22.49
3	s27833.1	18533662	0.77	0.87	0.75	0.87	0.83	0.24	0.75	0.79	27.72
3	s56018.1	18569142	0.66	0.66	0.74	0.90	0.64	0.22	0.62	0.80	24.13
3	OAR3_29960338.1	27732721	0.77	0.93	0.38	0.38	0.93	0.68	0.85	0.56	25.58
3	OAR3_34957910.1	32617818	0.75	0.83	0.79	0.75	0.76	0.70	0.83	0.83	30.09
3	s62202.1	37479731	0.36	0.83	0.66	0.86	0.84	0.25	0.40	0.83	22.72
3	OAR3_92984654.1	87822190	0.51	0.46	0.83	0.67	0.63	0.72	0.58	0.71	23.64

3	OAR3_96210394.1	90590980	0.75	0.50	0.66	0.62	0.70	0.49	0.80	0.66	23.90
3	s12587.1	93343121	0.77	0.64	0.69	0.46	0.70	0.37	0.80	0.84	24.37
3	OAR3_113978591.1	107081459	0.68	0.68	0.66	0.58	0.69	0.56	0.68	0.51	23.16
3	s53492.1	134161083	0.75	0.81	0.63	0.66	0.82	0.53	0.81	0.49	25.72
3	OAR3_143671114.1	134479165	0.85	0.64	0.83	0.78	0.62	0.60	0.47	0.89	26.88
3	OAR3_155880429.1	145971936	0.68	0.75	0.42	0.54	0.75	0.41	0.75	0.65	22.36
3	OAR3_163747812.1	153184360	0.72	0.55	0.77	0.79	0.50	0.51	0.52	0.61	22.88
3	OAR3_195793605_X.1	181785624	0.77	0.82	0.38	0.52	0.80	0.41	0.53	0.76	22.69
3	OAR3_195832851.1	181818709	0.77	0.82	0.41	0.52	0.80	0.41	0.50	0.71	22.41
3	OAR3_211975263.1	196781108	0.59	0.87	0.63	0.71	0.75	0.48	0.77	0.71	25.67
3	s03142.1	217226491	0.81	0.85	0.57	0.73	0.73	0.25	0.77	0.31	22.85
4	OAR4_4320614.1	4142006	0.64	0.85	0.63	0.64	0.73	0.15	0.79	0.71	23.43
4	OAR4_8579647.1	8554085	0.85	0.45	0.91	0.60	0.92	0.32	0.60	0.63	24.44
4	OAR4_14093754.1	13881724	0.70	0.79	0.74	0.46	0.80	0.50	0.79	0.63	25.28
4	s70608.1	14117077	0.77	0.96	0.77	0.82	0.96	0.31	0.75	0.91	29.93
4	s29388.1	33686327	0.72	0.72	0.63	0.70	0.73	0.38	0.53	0.72	23.55
4	OAR4_38097706.1	36117155	0.86	1.00	0.71	0.92	0.91	0.62	0.23	0.81	28.92
4	s32219.1	48304254	0.55	0.38	0.51	0.93	0.34	0.59	0.89	0.98	23.88
4	OAR4_60844851.1	57694339	0.68	0.93	0.88	0.85	0.51	0.27	0.84	0.81	27.37
4	OAR4_60864815.1	57713493	0.56	0.85	0.77	0.71	0.37	0.12	0.74	0.84	22.55
4	OAR4_62927453.1	59566544	0.61	0.37	0.61	0.77	0.60	0.61	0.64	0.72	22.48
4	OAR4_68066724.1	64328408	0.68	0.94	0.59	0.21	0.66	0.79	0.62	0.69	24.01
4	OAR4_94479590.1	88983257	0.89	0.61	0.48	0.90	0.72	0.66	0.61	0.91	27.28
5	s53436.1	13905219	0.59	0.92	0.54	0.41	0.85	0.65	0.77	0.71	25.37
5	OAR5_19894072.1	17340888	0.68	0.66	0.54	0.44	0.88	0.60	0.98	0.58	24.89
5	s71913.1	18245590	0.61	0.78	0.76	0.76	0.76	0.18	0.72	0.81	24.88

5	OAR5_27241937.1	24339868	0.87	0.98	0.66	0.65	0.86	0.56	0.81	0.71	29.22
5	OAR5_29603897.1	26619110	0.92	0.62	0.51	0.86	0.82	0.59	0.30	0.89	25.61
5	s63116.1	42948823	0.82	0.71	0.62	0.64	0.75	0.69	0.66	0.65	26.15
5	OAR5_47016261.1	42990841	0.81	0.78	0.57	0.59	0.79	0.74	0.69	0.60	26.17
5	OAR5_64198977.1	58379550	0.55	0.47	0.44	0.75	0.75	0.80	0.52	0.62	22.33
5	OAR5_67400660.1	61248799	0.59	0.59	0.83	0.87	0.38	0.75	0.91	0.67	26.55
5	OAR5_67426945.1	61268389	0.36	0.73	0.77	0.87	0.38	0.69	0.91	0.67	25.29
5	OAR5_67605574.1	61448352	0.72	0.55	0.63	0.73	0.62	0.78	0.87	0.69	26.35
5	OAR5_67725472_X.1	61562358	0.81	0.72	0.71	0.78	0.58	0.90	0.83	0.87	30.01
5	s01547.1	63523431	0.89	0.55	0.84	0.60	0.62	0.60	0.60	0.78	25.81
5	s01265.1	63546724	0.87	0.87	0.86	0.77	0.61	0.69	0.87	0.73	30.51
5	s75037.1	67865996	0.83	0.96	0.83	0.70	0.90	0.62	0.44	0.32	26.56
5	s34559.1	68392726	0.83	0.77	0.72	0.75	0.78	0.74	0.47	0.10	24.09
6	OAR6_17199790.1	14419851	0.70	0.50	0.70	0.66	0.49	0.34	0.91	0.77	23.17
6	OAR6_33210385.1	29372590	0.87	0.77	0.40	0.53	0.87	0.59	0.50	0.56	23.18
6	OAR6_33453920.1	29609130	0.83	0.94	0.71	1.00	0.98	0.59	0.47	0.93	31.17
6	s37521.1	34965214	0.74	0.77	0.54	0.85	0.19	0.54	0.49	0.80	22.40
6	OAR6_64114132.1	58220575	0.58	0.63	0.60	0.59	0.49	0.52	0.77	0.86	23.14
6	OAR6_68186441.1	62069219	0.87	0.79	0.48	0.66	0.74	0.69	0.50	0.82	25.97
6	OAR6_69728277.1	63262102	0.72	0.55	0.84	0.88	0.48	0.68	0.60	0.34	23.61
6	OAR6_93791340.1	85600434	0.45	0.58	0.60	0.55	0.75	0.38	0.80	0.85	22.49
6	s15912.1	114631910	0.53	0.87	0.79	0.63	0.76	0.77	0.92	0.52	27.52
7	OAR7_21705646.1	20823806	0.53	0.77	0.62	0.74	0.70	0.73	0.61	0.79	25.76
7	OAR7_33605191.1	29834504	0.81	0.66	0.63	0.66	0.28	0.78	0.64	0.60	23.51
7	OAR7_36652030.1	32477867	0.77	0.87	0.47	0.74	0.85	0.87	0.89	0.53	28.64
7	OAR7_60254585.1	54439369	0.87	0.87	0.86	0.86	0.83	0.82	0.81	0.79	33.02

7	OAR7_62972902.1	56858176	0.74	0.74	0.58	0.72	0.72	0.63	0.15	0.71	22.80
7	OAR7_63692612.1	57764872	0.64	0.67	0.73	0.46	0.55	0.78	0.55	0.69	23.49
7	OAR7_63745942.1	57816492	0.69	0.67	0.80	0.49	0.82	0.75	0.81	0.81	27.83
7	OAR7_71146927.1	64945291	0.79	0.69	0.74	0.71	0.78	0.10	0.60	0.63	22.91
7	OAR7_71507644.1	65314697	0.83	0.61	0.60	0.72	0.84	0.34	0.61	0.77	24.47
7	OAR7_91765475.1	84304384	0.74	0.74	0.48	0.72	0.75	0.67	0.74	0.61	25.50
7	OAR7_96995223.1	89156865	0.67	0.94	0.69	0.85	0.67	0.16	0.67	0.69	24.62
7	OAR7_97009654.1	89171610	0.75	0.96	0.76	0.65	0.74	0.01	0.64	0.76	24.17
8	OAR9_760.1	2459	0.77	0.58	0.48	0.68	0.80	0.49	0.38	0.85	22.81
8	OAR8_30023587.1	27526693	0.34	0.71	0.44	0.68	0.73	0.69	0.87	0.73	23.90
8	OAR8_32693991.1	30169514	0.66	0.66	0.60	0.64	0.64	0.58	0.66	0.52	22.75
8	OAR8_48645293.1	45154596	0.80	0.69	0.69	0.68	0.62	0.53	0.75	0.43	24.12
8	s34333.1	47947284	0.82	0.50	0.28	0.43	0.85	0.66	0.66	0.77	22.47
8	OAR8_55339073.1	51474008	0.64	0.69	0.35	0.55	0.84	0.77	0.77	0.63	24.19
8	OAR8_60543455.1	56536992	0.96	0.89	0.64	0.38	0.90	0.76	0.81	0.52	27.96
8	s61733.1	56613713	0.73	0.84	0.42	0.54	0.87	0.46	0.89	0.74	25.56
8	OAR8_60730321.1	56705959	0.64	0.55	0.51	0.79	0.87	0.74	0.29	0.71	23.38
8	OAR8_61394328.1	57306669	0.53	0.70	0.64	0.30	0.88	0.73	0.39	0.83	22.87
8	s66468.1	68307147	0.57	0.77	0.53	0.43	0.57	0.53	0.93	0.65	22.79
8	OAR8_74032292.1	69011617	0.66	0.78	0.56	0.75	0.76	0.74	0.72	0.68	26.76
8	s56071.1	72441567	0.75	0.57	0.72	0.34	0.76	0.81	0.61	0.70	24.60
8	s50153.1	74816521	0.40	0.87	0.63	0.73	0.98	0.59	0.72	0.76	26.77
8	s48807.1	85410438	0.36	0.71	0.68	0.71	0.51	0.69	0.51	0.87	23.15
9	s05716.1	17269965	0.63	0.74	0.65	0.42	0.82	0.08	0.76	0.87	22.50
9	OAR9_28653165.1	27379811	0.61	0.69	0.83	0.64	0.70	0.52	0.82	0.38	24.16
9	OAR9_28704738_X.1	27431994	0.64	0.56	0.86	0.49	0.66	0.48	0.84	0.35	22.45

9	OAR9_37300722.1	35414177	0.59	0.76	0.71	0.61	0.78	0.52	0.74	0.68	25.26
9	OAR9_71011585.1	66993812	0.82	0.79	0.74	0.79	0.31	0.84	0.69	0.69	27.07
9	OAR9_81293209.1	76704045	0.72	0.85	0.63	0.68	0.60	0.49	0.82	0.85	26.48
9	OAR9_92965915.1	87717571	0.89	0.66	0.61	0.57	0.74	0.37	0.70	0.72	24.32
10	s63205.1	9909833	0.54	0.87	0.77	0.71	0.29	0.57	0.75	0.86	25.14
10	OAR10_24992778.1	25086996	0.49	0.70	0.49	0.33	0.64	0.78	0.83	0.98	24.20
10	OAR10_28516285.1	28502457	0.59	0.89	0.74	0.82	0.73	0.55	0.57	0.89	27.35
10	OAR10_29223007.1	29188403	0.70	0.92	0.88	0.63	0.86	0.97	0.98	0.72	32.80
10	s02289.1	29202499	0.75	0.75	0.69	0.51	0.75	0.66	0.57	0.74	25.44
10	OAR10_50832739.1	50003887	0.82	0.36	0.88	0.40	0.94	0.78	0.93	0.44	26.36
10	OAR10_51830650.1	50876668	0.83	0.72	0.69	0.62	0.84	0.15	0.83	0.77	25.26
10	OAR10_59202250.1	58028982	0.36	0.50	0.80	0.95	0.66	0.63	0.82	0.65	25.07
10	OAR10_65844667.1	63849945	0.89	0.87	0.69	0.98	0.92	0.95	0.94	0.98	35.75
10	OAR10_69217913.1	66966639	0.87	0.82	0.71	0.86	0.88	0.58	0.79	0.87	30.89
10	s52066.1	76379406	0.54	0.67	0.68	0.38	0.71	0.67	0.70	0.63	22.89
10	s52900.1	85889796	0.70	0.82	0.72	0.85	0.78	0.78	0.87	0.84	30.82
11	s29885.1	18759252	0.59	0.67	0.60	0.88	0.82	0.87	0.84	0.51	27.52
11	s17310.1	19385272	0.84	0.73	0.83	0.43	0.21	0.47	0.89	0.70	23.68
11	s11660.1	41465606	0.19	0.76	0.71	0.36	0.55	0.82	0.69	0.79	22.34
11	OAR11_46036985.1	43267998	0.33	0.61	0.70	0.63	0.65	0.72	0.61	0.69	22.64
11	s27794.1	43375182	0.67	0.79	0.60	0.77	0.76	0.42	0.73	0.45	23.87
11	s26322.1	52708895	0.74	0.80	0.72	0.48	0.60	0.59	0.47	0.56	22.81
12	s60216.1	4813704	0.79	0.76	0.54	0.67	0.80	0.61	0.76	0.33	24.32
12	OAR12_15898442.1	13230931	0.75	0.61	0.56	0.49	0.59	0.78	0.64	0.93	24.94
12	OAR12_20263016.1	17379635	0.85	0.87	0.86	0.58	0.65	0.58	0.74	0.71	27.99
12	OAR12_20919634.1	18039881	0.63	0.73	0.71	0.70	0.73	0.38	0.73	0.46	23.25

12	s48775.1	38966266	0.79	0.72	0.69	0.49	0.80	0.59	0.49	0.96	25.89
12	OAR12_43408733.1	39002907	0.79	0.72	0.69	0.49	0.80	0.56	0.49	0.96	25.72
12	s27291.1	51724365	0.60	0.64	0.88	0.65	0.64	0.65	0.83	0.13	23.35
12	s65734.1	68272648	0.68	0.64	0.77	0.34	0.70	0.53	0.75	0.96	25.04
12	OAR12_81633377.1	74114369	0.61	0.62	0.74	0.65	0.56	0.71	0.85	0.74	25.81
12	s01191.1	74167822	0.27	0.73	0.75	0.51	0.65	0.66	0.93	0.62	23.73
13	OAR13_24394537.1	21881830	0.68	0.87	0.62	0.62	0.62	0.18	0.81	0.89	24.33
13	OAR13_24544219.1	22028605	0.64	0.87	0.81	0.75	0.62	0.11	0.85	0.89	25.84
13	OAR13_24835229.1	22312756	0.66	0.79	0.80	0.67	0.87	0.52	0.89	0.53	27.24
13	OAR13_29815318.1	26939029	0.80	0.48	0.77	0.57	0.46	0.60	0.82	0.58	23.55
13	OAR13_29868000.1	26997342	0.85	0.55	0.84	0.65	0.54	0.68	0.87	0.63	26.69
13	OAR13_60855392.1	55970160	0.82	0.46	0.77	0.74	0.78	0.55	0.31	0.82	24.32
13	OAR13_73412719.1	68241391	0.71	0.74	0.69	0.65	0.75	0.63	0.33	0.74	24.36
13	OAR13_73486401.1	68306113	0.71	0.74	0.69	0.65	0.75	0.51	0.36	0.64	23.23
13	OAR13_80086479.1	74377028	0.77	0.79	0.57	0.62	0.85	0.75	0.79	0.38	25.95
13	OAR13_80474846.1	74772124	0.89	0.91	0.95	0.79	0.85	0.81	0.91	0.81	34.31
13	OAR13_80560030.1	74859132	0.84	0.87	0.88	0.73	0.71	0.75	0.87	0.73	31.08
13	s75919.1	78004271	0.89	0.93	0.83	0.76	0.96	0.75	0.93	0.77	33.59
14	OAR14_15210238.1	14953941	0.65	0.68	0.58	0.54	0.66	0.64	0.68	0.68	23.50
14	OAR14_15705649_X.1	15438181	0.89	0.64	0.46	0.43	0.54	0.81	0.61	0.66	23.33
14	s45533.1	33094234	0.77	0.65	0.58	0.57	0.77	0.74	0.74	0.58	25.21
15	s74830.1	7575809	0.37	0.70	0.44	0.76	0.67	0.46	0.74	0.81	22.37
15	OAR15_34772476.1	33118615	0.66	0.77	0.54	0.98	0.86	0.68	0.47	0.63	26.11
15	OAR15_35681300.1	33931575	0.42	0.77	0.75	0.57	0.77	0.55	0.74	0.52	23.41
16	OAR16_9610934.1	8760018	0.64	0.52	0.51	0.65	0.78	0.69	0.52	0.76	23.25
16	OAR16_19296989.1	17491152	0.72	0.53	0.70	0.70	0.55	0.45	0.72	0.72	23.49

16	OAR16_60658467.1	55603528	0.74	0.85	0.61	0.32	0.90	0.41	0.92	0.40	23.78
16	s36879.1	56455419	0.18	0.59	0.69	0.71	0.96	0.44	0.64	0.72	22.32
16	s69802.1	56577830	0.55	0.62	0.71	0.60	0.98	0.76	0.87	0.74	27.83
17	s74460.1	9546160	0.79	0.87	0.62	0.76	0.73	0.69	0.79	0.73	28.58
17	OAR17_10638840.1	9579009	0.72	0.50	0.62	0.64	0.25	0.75	0.84	0.69	23.16
17	OAR17_31805652.1	29036982	0.60	0.84	0.66	0.41	0.53	0.62	0.77	0.61	23.18
17	OAR17_31867704.1	29099210	0.63	0.75	0.76	0.24	0.55	0.64	0.66	0.74	22.92
17	OAR17_38448146.1	35423021	0.54	0.59	0.57	0.68	0.71	0.68	0.79	0.71	24.50
18	OAR18_49661899.1	46642071	0.58	0.84	0.71	0.80	0.88	0.82	0.36	0.34	24.88
18	OAR18_61310611.1	57472621	0.60	0.49	0.91	0.60	0.74	0.65	0.68	0.78	25.64
18	OAR18_70842719.1	66756616	0.81	0.66	0.76	0.69	0.76	0.39	0.67	0.75	25.67
18	s48176.1	67289143	0.82	0.87	0.83	0.24	0.73	0.65	0.82	0.57	26.26
19	s29913.1	916549	0.21	0.32	0.74	0.75	0.80	0.71	0.68	0.78	22.80
19	OAR19_48620861.1	46183134	0.84	0.84	0.88	0.82	0.83	0.81	0.82	0.82	32.65
20	OAR20_10249189.1	10109112	0.89	0.48	0.83	0.88	0.31	0.45	0.59	0.68	23.68
20	s46840.1	36717434	0.76	0.60	0.60	0.59	0.85	0.45	0.69	0.81	24.79
20	OAR20_54293452.1	49822123	0.89	0.86	0.30	0.50	0.78	0.75	0.77	0.64	25.59
21	s37779.1	31372462	0.87	0.84	0.33	0.28	0.47	0.42	0.89	0.89	22.70
22	OAR22_6293170.1	5271128	0.69	0.68	0.62	0.80	0.63	0.62	0.51	0.46	22.94
22	OAR22_25489482.1	21644518	0.72	0.85	0.61	0.63	0.92	0.68	0.66	0.58	26.66
22	OAR22_36996817.1	32194893	0.50	0.52	0.71	0.43	0.80	0.68	0.84	0.81	24.72
22	OAR22_37025442.1	32223607	0.47	0.52	0.74	0.40	0.46	0.75	0.84	0.84	23.24
23	OAR23_21580936.1	20363654	0.94	0.30	0.67	0.46	0.54	0.62	0.79	0.80	23.71
23	OAR23_48878158.1	46167308	0.54	0.70	0.69	0.57	0.87	0.53	0.45	0.65	22.97
23	s04819.1	47503671	0.77	0.41	0.77	0.64	0.42	0.65	0.66	0.65	23.03
23	s43150.1	47511740	0.77	0.41	0.77	0.64	0.42	0.65	0.66	0.65	23.03

23	OAR23_59952060.1	56300415	0.60	0.87	0.36	0.70	0.90	0.56	0.53	0.54	22.98
24	s16016.1	24591560	0.49	0.74	0.74	0.93	0.90	0.38	0.85	0.96	28.37
24	s00643.1	24846522	0.94	1.00	0.43	0.78	0.70	0.35	0.39	0.89	25.32
24	OAR24_27348134_X.1	24859938	0.72	0.85	0.54	0.78	0.72	0.05	0.39	0.93	22.32
24	OAR24_27411708.1	24925070	0.70	0.91	0.64	0.74	0.83	0.53	0.75	0.93	28.79
24	s07554.1	28405516	0.78	0.67	0.60	0.79	0.73	0.75	0.58	0.78	26.82
24	s17819.1	37202588	0.64	0.79	0.52	0.79	0.71	0.75	0.36	0.63	23.92
25	s09722.1	9173663	0.89	0.77	0.45	0.20	0.96	0.35	0.68	0.89	23.70
25	OAR25_15495515.1	15121093	0.66	0.72	0.64	0.60	0.54	0.48	0.72	0.58	22.58
25	s56125.1	39254408	0.82	0.69	0.68	0.31	0.75	0.66	0.69	0.82	25.36
25	s44634.1	40388416	0.53	0.68	0.54	0.81	0.84	0.71	0.81	0.56	25.51
25	OAR25_43417329.1	41096160	0.42	0.72	0.56	0.39	0.80	0.56	0.77	0.83	23.03
26	OAR26_12979568.1	10434838	0.74	0.74	0.69	0.44	0.72	0.52	0.71	0.46	23.09
26	OAR26_38406348_X.1	33708849	0.50	0.55	0.60	0.50	0.87	0.81	0.77	0.84	25.40

Notes: Significant SNPs located within merged genomic regions are colored “red”.

Table S6. Highly differentiated genomic regions between Bhyanglung and low-land sheep breeds

CHR	Start(bp)	End(bp)	Length	Number of SNPs	Max Di	Min Di	Mean Di
1	99931717	100185774	254057	2	37.45	29.80	33.63
2	110291246	110743186	451940	2	26.51	26.40	26.45
2	220053519	220103004	49485	2	28.66	23.85	26.26
2	237813990	237822440	8450	2	30.57	26.58	28.58
3	41551185	41793029	241844	2	32.75	23.03	27.89
3	92401501	92497164	95663	2	25.82	23.19	24.50
3	93522006	93725974	203968	2	22.81	22.54	22.68
4	13881724	14355529	473805	2	25.51	24.22	24.86
4	48201750	48669962	468212	7	48.98	25.82	36.44
5	51535099	51587928	52829	2	26.66	22.80	24.73
6	94639943	94749679	109736	2	38.65	23.13	30.89
7	15988141	15995141	7000	2	31.77	21.85	26.81
7	57692469	57915106	222637	5	54.92	28.48	45.42
8	38108511	38129829	21318	2	32.88	30.70	31.79
10	29054709	29806294	751585	5	50.62	23.19	35.21
10	30618253	30700853	82600	3	25.30	22.58	23.68
11	7858660	7898325	39665	2	24.28	24.26	24.27
11	19223203	19397667	174464	3	29.32	24.31	27.33
11	22780752	22814442	33690	2	37.33	36.98	37.15
11	26421905	26872280	450375	2	24.52	22.51	23.52
12	38923821	39002907	79086	2	30.45	25.96	28.20
13	32586633	32625552	38919	2	22.88	21.82	22.35
16	30655866	30720540	64674	3	29.47	25.57	27.37
16	70710282	70922464	212182	3	26.97	24.74	25.91
19	34817411	34838172	20761	2	24.77	22.88	23.82
20	34168829	34256995	88166	2	41.67	30.62	36.15
22	42969386	43182266	212880	2	25.21	23.30	24.26
23	32282622	32466427	183805	4	33.48	23.28	27.35

Table S7. Highly differentiated genomic regions between Tibetan and low-land sheep breeds

CHR	Start(bp)	End(bp)	Length	Number of SNPs	Max Di	Min Di	Mean Di
1	73928927	74316221	387294	2	25.28	22.85	24.06
1	99931717	100185774	254057	2	42.20	26.57	34.38
1	183336939	183575060	238121	3	37.38	28.75	33.04
2	100199475	100364649	165174	2	34.10	26.90	30.50
2	135304995	135636519	331524	3	39.22	23.04	29.38
2	237813990	237822440	8450	2	29.66	28.21	28.93
3	39851694	40956953	1105259	10	41.88	22.64	32.98
3	79473981	79511180	37199	2	25.09	22.91	24.00
4	48304254	48669962	365708	3	50.74	28.75	36.08
4	87572495	87649810	77315	3	46.37	37.02	41.57
5	15912332	16520048	607716	4	27.19	25.12	25.88
5	19778519	19986771	208252	2	26.23	23.24	24.73
5	51535099	51587928	52829	2	33.53	25.12	29.32
7	42463209	42551974	88765	2	40.15	38.07	39.11
7	57631745	57915106	283361	5	48.98	23.23	39.27
8	38046068	38129829	83761	3	35.43	32.50	33.70
9	77297358	77756891	459533	2	27.53	24.84	26.19
10	29054709	29806294	751585	5	49.84	27.49	38.79
10	30674002	30700853	26851	2	27.22	25.29	26.25
11	19385272	19397667	12395	2	36.00	26.64	31.32
11	22736347	22814442	78095	3	31.59	24.80	29.11
12	38843578	39002907	159329	3	38.33	29.85	34.67
12	64847102	64870223	23121	2	25.01	24.28	24.64
13	78004271	78420224	415953	2	28.94	27.18	28.06
15	31492057	31723078	231021	2	26.54	23.77	25.16
15	72707350	72747023	39673	2	33.05	23.58	28.31
16	30655866	30847022	191156	3	24.92	24.08	24.50
16	70900966	70922464	21498	2	27.03	26.23	26.63
17	1440961	1509630	68669	2	38.34	23.81	31.08
17	12319170	12471246	152076	2	25.65	25.49	25.57
17	53470559	53511490	40931	3	33.00	28.51	30.85
18	19342316	19399095	56779	2	28.51	24.62	26.57
18	44805751	44858471	52720	2	26.50	25.40	25.95
20	34168829	34256995	88166	2	33.58	25.06	29.32
22	42969386	43123707	154321	2	28.64	25.17	26.90
23	25919106	25972928	53822	2	44.46	27.00	35.73

Table S8. Highly differentiated genomic regions between Changthangi and low-land sheep breeds

CHR	Start(bp)	End(bp)	Length	Number of SNPs	Max Di	Min Di	Mean Di
1	99931717	100185774	254057	2	32.27	27.08	29.67
1	247826668	248275678	449010	2	32.97	22.69	27.83
2	30919873	31018254	98381	2	28.04	23.58	25.81
2	81785728	82346323	560595	3	29.85	21.76	25.78
2	164516447	164594319	77872	2	25.41	23.68	24.55
2	232414241	232485668	71427	2	33.66	22.64	28.15
2	237813990	237822440	8450	2	28.75	28.75	28.75
3	62015360	62044832	29472	2	34.87	30.83	32.85
3	108409583	108434471	24888	3	30.82	21.58	25.01
3	162819252	163307018	487766	2	31.67	27.83	29.75
3	165413630	165785278	371648	2	26.14	22.29	24.22
3	178423049	178743725	320676	4	28.71	21.20	24.12
3	180817708	180839858	22150	2	25.14	21.55	23.35
4	48304254	48669962	365708	3	36.44	21.14	28.51
4	75968047	76007878	39831	2	31.74	24.41	28.08
4	87572495	87649810	77315	2	26.86	23.30	25.08
5	40487686	40713566	225880	2	27.30	21.84	24.57
5	51587928	51843084	255156	2	25.43	24.71	25.07
7	42463209	42551974	88765	2	25.13	21.25	23.19
7	57692469	57915106	222637	5	51.10	34.34	43.55
7	79817839	79831939	14100	2	23.43	21.51	22.47
7	89286934	89519883	232949	2	21.75	21.34	21.54
8	10725738	10820850	95112	2	26.15	22.85	24.50
9	61628833	61661127	32294	2	27.38	21.01	24.20
9	77297358	77756891	459533	2	33.76	33.58	33.67
10	29054709	29502667	447958	4	35.75	21.85	29.06
11	19223203	19397667	174464	3	29.65	24.63	27.03
11	22780752	22814442	33690	2	25.95	25.60	25.78
11	49142365	49193942	51577	2	23.84	22.63	23.24
12	38843578	39002907	159329	3	30.68	24.02	26.28
15	42070930	42207222	136292	2	23.01	22.69	22.85
16	34279856	34315060	35204	2	32.04	25.95	28.99
17	21434752	21454166	19414	2	29.33	24.92	27.13
18	19342316	19399095	56779	2	25.35	23.92	24.64
20	34168829	34256995	88166	2	32.03	22.65	27.34
24	9627147	9721533	94386	3	31.96	22.57	26.52

Table S9. Highly differentiated genomic regions between Baruwal and low-land sheep breeds

CHR	Start(bp)	End(bp)	Length	Number of SNPs	Max Di	Min Di	Mean Di
1	249966956	250456371	489415	2	28.44	24.19	26.31
2	48731082	48762835	31753	2	24.70	23.46	24.08
2	52177597	52218919	41322	2	27.01	22.89	24.95
2	100199475	100364649	165174	2	26.28	24.65	25.46
2	139846639	140158515	311876	2	22.83	22.38	22.61
2	145693768	145745763	51995	2	26.25	24.96	25.61
2	146734798	146754173	19375	2	27.33	25.57	26.45
3	18533662	18569142	35480	2	27.72	24.13	25.92
3	134161083	134479165	318082	2	26.88	25.72	26.30
3	181785624	181818709	33085	2	22.69	22.41	22.55
4	13881724	14117077	235353	2	29.93	25.28	27.61
4	57694339	57713493	19154	2	27.37	22.55	24.96
5	42948823	42990841	42018	2	26.17	26.15	26.16
5	61248799	61562358	313559	4	30.01	25.29	27.05
5	63523431	63546724	23293	2	30.51	25.81	28.16
6	29372590	29609130	236540	2	31.17	23.18	27.18
7	57764872	57816492	51620	2	27.83	23.49	25.66
7	64945291	65314697	369406	2	24.47	22.91	23.69
7	89156865	89171610	14745	2	24.62	24.17	24.40
8	56536992	56705959	168967	3	27.96	23.38	25.63
9	27379811	27431994	52183	2	24.16	22.45	23.31
10	29188403	29202499	14096	2	32.80	25.44	29.12
11	43267998	43375182	107184	2	23.87	22.64	23.26
12	38966266	39002907	36641	2	25.89	25.72	25.81
12	74114369	74167822	53453	2	25.81	23.73	24.77
13	21881830	22312756	430926	3	27.24	24.33	25.80
13	26939029	26997342	58313	2	26.69	23.55	25.12
13	68241391	68306113	64722	2	24.36	23.23	23.80
13	74377028	74859132	482104	3	34.31	25.95	30.45
14	14953941	15438181	484240	2	23.50	23.33	23.41
16	56455419	56577830	122411	2	27.83	22.32	25.08
17	9546160	9579009	32849	2	28.58	23.16	25.87
17	29036982	29099210	62228	2	23.18	22.92	23.05
22	32194893	32223607	28714	2	24.72	23.24	23.98
23	47503671	47511740	8069	2	23.03	23.03	23.03
24	24591560	24925070	333510	4	28.79	22.32	26.20

Table S10. Information of samples used in analysis of *FGF-7* sequence

**Sample ID**	**Population**	**Origin**
OARI_ZB08	Tibetan	China
OARI_ZD11	Tibetan	China
OARI_CHA02	Changthangi	India
OARI_CHA05	Changthangi	India
OARI_BGE2	Bangladeshi	Bangladesh
OARI_BGE4	Bangladeshi	Bangladesh
OARI_GAR14	Garole	
OARI_GAR4	Indian Garole	Indian
OARI_GUR4	Garut	Indonesia
OARI_GUR5	Garut	Indonesia
OARI_SUM2	Sumatra	Indonesia
OARI_SUM7	Sumatra	Indonesia

Table S11. SNPs identified in *FGF-7* gene

Chr	Position	A1	Freq (High)	Freq (Others)	A2	P value	Type
7	57775018	A	1.000	0.188	G	2.24E-04	downstream
7	57775068	A	0.667	0.938	T	1.69E-01	downstream
7	57775168	A	1.000	0.313	G	1.97E-03	downstream
7	57775230	A	1.000	0.313	T	1.97E-03	downstream
7	57775389	T	0.625	0.813	C	3.62E-01	downstream
7	57775478	C	0.625	0.125	A	2.07E-02	downstream
7	57775635	T	0.667	0.938	C	1.69E-01	downstream
7	57775680	A	0.625	0.125	G	2.07E-02	downstream
7	57776040	T	0.625	0.125	C	2.07E-02	downstream
7	57776043	G	0.625	0.125	A	2.07E-02	downstream
7	57776205	G	0.625	0.125	A	2.07E-02	downstream
7	57776247	C	0.625	0.125	T	2.07E-02	downstream
7	57776248	A	0.625	0.125	G	2.07E-02	downstream
7	57776371	T	0.625	0.938	C	9.09E-02	downstream
7	57776412	T	0.625	0.125	G	2.07E-02	downstream
7	57776523	C	0.625	0.125	T	2.07E-02	downstream
7	57776654	C	0.625	0.125	T	2.07E-02	downstream
7	57776799	G	0.625	0.938	C	9.09E-02	downstream
7	57776801	T	0.625	0.125	C	2.07E-02	downstream
7	57776868	A	0.625	0.125	T	2.07E-02	downstream
7	57776969	G	0.625	0.125	A	2.07E-02	downstream
7	57777259	G	0.625	0.071	A	1.09E-02	downstream
7	57777431	C	0.625	0.125	T	2.07E-02	downstream
7	57777565	A	0.667	0.125	C	2.54E-02	downstream
7	57777614	G	1.000	0.250	A	1.35E-03	downstream
7	57778098	G	0.625	0.875	T	2.89E-01	downstream
7	57778245	G	1.000	0.286	A	1.69E-03	downstream
7	57778594	A	1.000	0.250	G	1.35E-03	downstream
7	57778598	A	0.625	0.125	C	2.07E-02	downstream
7	57778602	G	0.625	0.125	A	2.07E-02	downstream
7	57778788	T	0.625	0.125	C	2.07E-02	downstream
7	57779093	T	0.625	0.125	C	2.07E-02	downstream
7	57779150	G	0.625	0.125	C	2.07E-02	downstream
7	57779453	T	0.625	0.125	C	2.07E-02	downstream
7	57779927	G	0.625	0.125	A	2.07E-02	downstream
7	57780357	T	0.625	0.125	G	2.07E-02	UTR3
7	57780557	T	0.625	0.125	C	2.07E-02	exonic
7	57780888	T	1.000	0.250	C	1.35E-03	intronic
7	57780917	T	0.625	0.875	G	2.89E-01	intronic
7	57780946	C	0.625	0.938	A	9.09E-02	intronic

7	57780971	C	0.625	0.938	T	9.09E-02	intronic
7	57781545	G	0.625	0.938	A	9.09E-02	intronic
7	57781719	A	0.625	0.125	G	2.07E-02	intronic
7	57782555	A	0.833	0.125	G	4.32E-03	intronic
7	57782869	G	0.625	0.125	A	2.07E-02	intronic
7	57782948	A	0.625	0.875	G	2.89E-01	intronic
7	57783024	T	0.625	0.125	G	2.07E-02	intronic
7	57783483	T	1.000	0.313	G	1.97E-03	intronic
7	57783679	A	0.625	0.875	G	2.89E-01	intronic
7	57783832	G	0.625	0.875	A	2.89E-01	intronic
7	57784005	G	0.625	0.875	C	2.89E-01	intronic
7	57784007	G	0.625	0.857	T	3.09E-01	intronic
7	57784047	G	0.625	0.125	A	2.07E-02	intronic
7	57784147	C	0.625	0.875	T	2.89E-01	intronic
7	57784599	A	0.625	0.125	G	2.07E-02	intronic
7	57784617	A	0.625	0.125	G	2.07E-02	intronic
7	57784734	A	0.625	0.875	G	2.89E-01	intronic
7	57784985	G	1.000	0.250	A	1.35E-03	intronic
7	57785176	A	0.625	0.875	G	2.89E-01	intronic
7	57785244	T	0.625	0.071	C	1.09E-02	intronic
7	57785507	G	0.625	0.875	A	2.89E-01	intronic
7	57785940	A	1.000	0.250	G	1.35E-03	intronic
7	57786301	T	0.625	0.875	G	2.89E-01	intronic
7	57786468	G	0.625	0.125	A	2.07E-02	intronic
7	57787035	A	0.625	0.875	T	2.89E-01	intronic
7	57787049	T	0.625	0.875	C	2.89E-01	intronic
7	57787297	T	0.625	0.875	C	2.89E-01	intronic
7	57787298	G	0.625	0.875	A	2.89E-01	intronic
7	57787393	T	0.625	0.875	C	2.89E-01	intronic
7	57788007	G	1.000	0.250	A	1.35E-03	intronic
7	57788150	G	0.625	0.875	A	2.89E-01	intronic
7	57788204	G	0.625	0.938	C	9.09E-02	intronic
7	57788328	A	0.625	0.125	C	2.07E-02	intronic
7	57788401	T	0.625	0.875	C	2.89E-01	intronic
7	57788520	G	0.625	0.143	A	5.24E-02	intronic
7	57788734	G	0.625	0.125	T	2.07E-02	intronic
7	57788781	T	0.625	0.875	G	2.89E-01	intronic
7	57788963	C	0.625	0.143	T	5.24E-02	intronic
7	57789030	A	0.625	0.125	C	2.07E-02	intronic
7	57789182	G	0.625	0.929	C	1.17E-01	intronic
7	57789241	A	1.000	0.250	G	1.35E-03	intronic
7	57789295	G	0.625	0.125	T	2.07E-02	intronic
7	57789795	A	1.000	0.313	G	1.97E-03	intronic

7	57789811	G	0.625	0.125	T	2.07E-02	intronic
7	57789934	A	0.625	0.875	C	2.89E-01	intronic
7	57789985	A	1.000	0.313	T	1.97E-03	intronic
7	57790004	A	1.000	0.313	G	1.97E-03	intronic
7	57790276	T	1.000	0.313	A	1.97E-03	intronic
7	57790587	C	0.625	0.250	A	9.94E-02	intronic
7	57790604	T	0.625	0.250	C	9.94E-02	intronic
7	57790889	A	0.625	0.938	G	9.09E-02	intronic
7	57790895	C	0.625	0.938	T	9.09E-02	intronic
7	57791333	T	1.000	0.313	A	1.97E-03	intronic
7	57791679	C	0.625	0.250	A	9.94E-02	intronic
7	57791794	A	0.625	0.250	T	9.94E-02	intronic
7	57791818	G	0.625	0.938	A	9.09E-02	intronic
7	57792079	A	1.000	0.875	C	5.36E-01	intronic
7	57792171	T	0.625	0.938	C	9.09E-02	intronic
7	57792535	G	0.625	0.938	A	9.09E-02	intronic
7	57792542	C	1.000	0.313	T	1.97E-03	intronic
7	57792555	C	0.625	0.125	A	2.07E-02	intronic
7	57792649	G	0.625	0.125	A	2.07E-02	intronic
7	57792875	G	0.625	0.938	T	9.09E-02	intronic
7	57792886	A	0.625	0.143	G	5.24E-02	intronic
7	57793139	A	0.625	0.125	G	2.07E-02	intronic
7	57793389	G	1.000	0.188	A	2.24E-04	intronic
7	57793461	T	0.625	0.125	A	2.07E-02	intronic
7	57793519	G	0.625	0.125	A	2.07E-02	intronic
7	57793528	A	1.000	0.188	G	2.24E-04	intronic
7	57793556	A	0.625	0.125	G	2.07E-02	intronic
7	57793618	C	0.625	0.125	G	2.07E-02	intronic
7	57793649	C	0.625	0.125	T	2.07E-02	intronic
7	57793928	T	0.625	0.125	C	2.07E-02	intronic
7	57793944	T	0.625	0.938	A	9.09E-02	intronic
7	57793957	A	0.625	0.125	G	2.07E-02	intronic
7	57794004	A	0.625	0.938	G	9.09E-02	intronic
7	57794078	C	1.000	0.875	A	5.36E-01	intronic
7	57794136	T	1.000	0.875	G	5.36E-01	intronic
7	57794198	C	1.000	0.875	T	5.36E-01	intronic
7	57794291	C	1.000	0.875	A	5.36E-01	intronic
7	57794483	A	1.000	0.875	G	5.36E-01	intronic
7	57794519	A	0.625	0.938	C	9.09E-02	intronic
7	57794525	A	1.000	0.875	G	5.36E-01	intronic
7	57794690	G	1.000	0.875	A	5.36E-01	intronic
7	57795314	G	0.625	0.938	T	9.09E-02	intronic
7	57795346	C	1.000	0.875	T	5.36E-01	intronic

7	57795437	C	1.000	0.875	T	5.36E-01	intronic
7	57795614	C	1.000	0.875	T	5.36E-01	intronic
7	57795660	C	1.000	0.875	T	5.36E-01	intronic
7	57796255	G	1.000	0.875	A	5.36E-01	intronic
7	57796591	A	1.000	0.875	C	5.36E-01	intronic
7	57796593	G	1.000	0.875	A	5.36E-01	intronic
7	57796612	G	1.000	0.875	A	5.36E-01	intronic
7	57796747	C	0.625	0.125	T	2.07E-02	intronic
7	57796808	T	0.625	0.938	C	9.09E-02	intronic
7	57797484	A	1.000	0.875	G	5.36E-01	intronic
7	57797489	G	1.000	0.313	A	1.97E-03	intronic
7	57797667	G	1.000	0.875	A	5.36E-01	intronic
7	57798166	G	1.000	0.875	C	5.36E-01	intronic
7	57798228	G	0.625	0.125	A	2.07E-02	intronic
7	57798240	A	1.000	0.875	G	5.36E-01	intronic
7	57798300	G	1.000	0.875	A	5.36E-01	intronic
7	57798572	A	0.625	0.071	C	1.09E-02	intronic
7	57798705	C	1.000	0.875	T	5.36E-01	intronic
7	57798831	G	0.625	0.125	C	2.07E-02	intronic
7	57798941	G	1.000	0.875	A	5.36E-01	intronic
7	57798991	A	1.000	0.875	G	5.36E-01	intronic
7	57799016	A	0.625	0.938	G	9.09E-02	intronic
7	57799134	T	0.625	0.125	G	2.07E-02	intronic
7	57799147	A	1.000	0.125	G	3.75E-04	intronic
7	57799170	C	1.000	0.313	T	1.97E-03	intronic
7	57799180	A	1.000	0.875	G	5.36E-01	intronic
7	57799527	A	1.000	0.313	G	1.97E-03	intronic
7	57799544	G	0.625	0.938	C	9.09E-02	intronic
7	57800056	A	1.000	0.875	T	5.36E-01	intronic
7	57800106	G	1.000	0.875	A	5.36E-01	intronic
7	57800141	G	1.000	0.875	A	5.36E-01	intronic
7	57800335	A	1.000	0.875	G	5.36E-01	intronic
7	57800770	A	1.000	0.875	G	5.36E-01	intronic
7	57800820	G	1.000	0.875	T	5.36E-01	intronic
7	57801015	A	0.625	0.125	G	2.07E-02	intronic
7	57801137	A	0.625	0.125	G	2.07E-02	intronic
7	57801469	C	0.625	0.938	T	9.09E-02	intronic
7	57801495	T	1.000	0.875	C	5.36E-01	intronic
7	57801908	C	0.625	0.929	T	1.17E-01	intronic
7	57802257	G	1.000	0.313	A	1.97E-03	intronic
7	57802419	C	0.625	0.938	T	9.09E-02	intronic
7	57802466	G	1.000	0.875	A	5.36E-01	intronic
7	57802578	G	0.625	0.938	C	9.09E-02	intronic

7	57802774	C	0.625	0.125	T	2.07E-02	intronic
7	57802876	A	0.625	0.813	C	3.62E-01	intronic
7	57803097	T	0.625	0.938	G	9.09E-02	intronic
7	57803109	A	0.625	0.125	T	2.07E-02	intronic
7	57803147	T	0.625	0.125	A	2.07E-02	intronic
7	57803439	T	1.000	0.375	C	6.43E-03	intronic
7	57803456	G	0.625	0.938	A	9.09E-02	intronic
7	57803552	T	0.625	0.813	C	3.62E-01	intronic
7	57803564	C	0.625	0.938	T	9.09E-02	intronic
7	57803661	G	1.000	0.313	A	1.97E-03	intronic
7	57803687	G	1.000	0.313	A	1.97E-03	intronic
7	57803711	G	1.000	0.313	A	1.97E-03	intronic
7	57803731	A	0.625	0.938	G	9.09E-02	intronic
7	57803782	C	0.625	0.125	T	2.07E-02	intronic
7	57804226	A	0.625	0.125	G	2.07E-02	intronic
7	57804316	G	0.625	0.938	A	9.09E-02	intronic
7	57804518	C	0.625	0.125	T	2.07E-02	intronic
7	57805065	G	0.625	0.125	T	2.07E-02	intronic
7	57805457	C	1.000	0.313	T	1.97E-03	intronic
7	57805498	C	0.625	0.938	T	9.09E-02	intronic
7	57805607	G	0.625	0.125	T	2.07E-02	intronic
7	57805651	T	0.625	0.125	C	2.07E-02	intronic
7	57806548	A	0.625	0.125	C	2.07E-02	intronic
7	57806869	C	0.625	0.938	T	9.09E-02	intronic
7	57806887	G	0.625	0.125	C	2.07E-02	intronic
7	57806993	T	0.625	0.125	C	2.07E-02	intronic
7	57807019	T	0.625	0.125	C	2.07E-02	intronic
7	57807165	T	0.625	0.125	A	2.07E-02	intronic
7	57807191	G	0.625	0.125	A	2.07E-02	intronic
7	57807201	T	0.625	0.125	C	2.07E-02	intronic
7	57807256	G	0.625	0.938	A	9.09E-02	intronic
7	57807270	A	0.625	0.125	C	2.07E-02	intronic
7	57807314	T	0.625	0.125	A	2.07E-02	intronic
7	57807324	G	1.000	0.188	A	2.24E-04	intronic
7	57807420	T	0.625	0.125	C	2.07E-02	intronic
7	57807424	T	0.625	0.938	A	9.09E-02	intronic
7	57807515	G	0.625	0.125	A	2.07E-02	intronic
7	57807541	A	0.625	0.125	C	2.07E-02	intronic
7	57807585	A	0.625	0.125	G	2.07E-02	intronic
7	57807908	T	1.000	0.250	C	1.35E-03	intronic
7	57807960	G	1.000	0.188	T	2.24E-04	intronic
7	57808082	G	1.000	0.875	A	5.36E-01	intronic
7	57809459	C	0.625	0.938	T	9.09E-02	intronic

7	57809897	C	0.625	0.938	A	9.09E-02	intronic
7	57809954	A	0.625	0.938	G	9.09E-02	intronic
7	57810413	A	0.625	0.143	G	5.24E-02	intronic
7	57810485	G	1.000	0.313	A	1.97E-03	intronic
7	57810967	G	1.000	0.250	T	1.35E-03	intronic
7	57811135	C	1.000	0.375	T	6.43E-03	intronic
7	57811164	G	1.000	0.313	A	1.97E-03	intronic
7	57811298	A	0.625	0.938	C	9.09E-02	intronic
7	57812157	C	1.000	0.250	T	1.35E-03	intronic
7	57812216	A	1.000	0.250	G	1.35E-03	intronic
7	57812355	G	1.000	0.250	A	1.35E-03	intronic
7	57812639	T	1.000	0.250	C	1.35E-03	intronic
7	57812772	C	1.000	0.250	T	1.35E-03	intronic
7	57813105	T	0.625	0.125	C	2.07E-02	intronic
7	57813614	T	0.625	0.125	C	2.07E-02	intronic
7	57814308	G	0.625	0.929	A	1.17E-01	intronic
7	57814325	T	0.625	0.500	C	6.75E-01	intronic
7	57814371	C	0.625	0.857	A	3.09E-01	intronic
7	57814439	G	0.625	0.643	A	1.00E+00	intronic
7	57814441	G	0.625	0.643	A	1.00E+00	intronic
7	57814533	C	0.625	0.750	T	6.47E-01	intronic
7	57814562	G	0.875	0.813	A	1.00E+00	intronic
7	57815226	T	0.750	0.125	A	4.74E-03	intronic
7	57815228	A	0.750	0.125	G	4.74E-03	intronic
7	57815376	G	0.750	1.000	A	1.01E-01	intronic
7	57815489	G	0.750	0.063	A	1.32E-03	intronic
7	57815570	C	0.750	1.000	A	1.01E-01	intronic
7	57816040	A	0.750	0.063	G	1.32E-03	intronic
7	57816492	T	0.750	0.063	C	1.32E-03	intronic
7	57816974	G	1.000	0.313	A	1.97E-03	intronic
7	57817884	A	0.750	0.063	G	1.32E-03	intronic
7	57817978	A	1.000	0.875	T	5.36E-01	intronic
7	57818293	A	0.750	0.063	G	1.32E-03	intronic
7	57818516	A	0.750	0.063	G	1.32E-03	intronic
7	57819556	A	0.750	1.000	G	1.01E-01	intronic
7	57819756	G	0.750	1.000	A	1.01E-01	intronic
7	57821506	A	1.000	0.063	T	1.22E-05	intronic
7	57821577	T	0.750	0.063	G	1.32E-03	intronic
7	57821717	T	1.000	0.250	C	1.35E-03	intronic
7	57821805	C	0.750	1.000	T	1.01E-01	intronic
7	57822203	C	1.000	0.875	T	5.36E-01	intronic
7	57822679	C	1.000	0.313	T	1.97E-03	intronic
7	57823409	C	0.750	0.063	T	1.32E-03	intronic

7	57823568	G	1.000	0.063	A	1.22E-05	intronic
7	57824220	G	0.750	1.000	C	1.01E-01	intronic
7	57824362	T	1.000	0.063	C	1.22E-05	intronic
7	57824506	C	1.000	0.063	T	1.22E-05	intronic
7	57824633	T	0.750	0.063	C	1.32E-03	intronic
7	57825084	T	0.750	0.000	G	3.75E-04	intronic
7	57825268	G	0.750	0.063	A	1.32E-03	intronic
7	57825328	T	1.000	0.875	C	5.36E-01	intronic
7	57825562	C	1.000	0.875	T	5.36E-01	intronic
7	57825695	T	0.750	0.063	A	1.32E-03	intronic
7	57826956	A	0.750	0.063	G	1.32E-03	intronic
7	57827390	C	0.750	0.000	T	3.75E-04	intronic
7	57827438	T	0.750	0.063	C	1.32E-03	intronic
7	57827549	C	0.750	1.000	T	1.01E-01	intronic
7	57827663	A	1.000	0.063	G	1.22E-05	intronic
7	57827783	G	0.750	0.063	A	1.32E-03	intronic
7	57827820	C	0.750	1.000	T	1.01E-01	intronic
7	57828090	T	1.000	0.063	C	1.22E-05	intronic
7	57828134	C	1.000	0.250	T	1.35E-03	intronic
7	57828245	G	0.750	0.063	C	1.32E-03	intronic
7	57828573	C	0.750	1.000	T	1.01E-01	intronic
7	57828582	T	1.000	0.875	G	5.36E-01	intronic
7	57829238	G	1.000	0.063	T	1.22E-05	intronic
7	57829330	T	0.750	1.000	A	1.01E-01	intronic
7	57831166	A	0.750	1.000	C	1.01E-01	intronic
7	57831958	T	0.750	0.063	C	1.32E-03	intronic
7	57832031	A	1.000	0.063	G	1.22E-05	intronic
7	57832174	A	1.000	0.063	C	1.22E-05	intronic
7	57832504	G	0.750	0.063	A	1.32E-03	intronic
7	57833558	G	1.000	0.063	A	1.22E-05	intronic
7	57833677	A	0.750	0.063	G	1.32E-03	intronic
7	57833838	C	1.000	0.063	T	1.22E-05	intronic
7	57835192	A	0.750	1.000	G	1.01E-01	intronic
7	57836987	A	0.750	1.000	G	1.01E-01	intronic
7	57837504	G	1.000	0.063	A	1.22E-05	intronic
7	57838457	T	1.000	0.875	A	5.36E-01	intronic
7	57838459	C	1.000	0.188	T	2.24E-04	intronic
7	57838485	T	0.750	1.000	C	1.01E-01	intronic
7	57838986	A	0.750	0.063	T	1.32E-03	intronic
7	57839255	T	1.000	0.063	G	1.22E-05	intronic
7	57839520	A	0.750	1.000	G	1.01E-01	intronic
7	57839819	T	0.750	1.000	C	1.01E-01	intronic
7	57840274	G	0.750	1.000	A	1.01E-01	intronic

7	57841088	A	1.000	0.750	C	2.62E-01	intronic
7	57842782	A	0.750	1.000	G	1.01E-01	upstream
7	57842788	T	0.750	1.000	C	1.01E-01	upstream
7	57842871	G	0.750	0.250	A	3.24E-02	upstream
7	57842893	C	0.750	0.063	T	1.32E-03	upstream
7	57843431	T	0.750	1.000	G	1.01E-01	upstream
7	57843456	G	0.750	0.063	A	1.32E-03	upstream
7	57843555	T	0.750	1.000	C	1.01E-01	upstream
**7**	**57843681**	**A**	**1.000**	**0.063**	**T**	**9.38E-05**	upstream
7	57843718	A	1.000	0.250	G	1.35E-03	upstream
7	57844175	C	0.750	0.063	T	1.32E-03	upstream
7	57844343	C	0.750	0.250	G	3.24E-02	upstream
7	57844418	G	0.750	0.250	A	3.24E-02	upstream
7	57844466	T	0.750	0.250	C	3.24E-02	upstream
7	57844474	T	0.750	0.250	C	3.24E-02	upstream
7	57844672	G	0.750	0.063	A	1.32E-03	upstream
7	57844777	A	0.750	0.063	G	1.32E-03	upstream
7	57844796	G	0.750	0.063	C	1.32E-03	upstream
7	57845008	C	0.750	0.250	T	3.24E-02	upstream
7	57845105	T	0.750	0.250	A	3.24E-02	upstream
7	57845189	G	1.000	0.250	A	1.35E-03	upstream
7	57845232	G	0.750	0.250	A	3.24E-02	upstream
7	57845269	T	0.750	0.250	C	3.24E-02	upstream
7	57845497	T	0.750	0.250	C	3.24E-02	upstream
7	57845863	A	0.750	0.250	C	3.24E-02	upstream
7	57845981	G	0.750	0.250	A	3.24E-02	upstream
7	57846117	C	1.000	0.250	T	1.35E-03	upstream
7	57846357	T	0.750	0.250	A	3.24E-02	upstream
7	57846413	T	1.000	0.813	C	5.26E-01	upstream
7	57846450	A	1.000	0.813	C	5.26E-01	upstream
7	57846495	A	0.750	1.000	G	1.01E-01	upstream

Notes: A1, dominant allele in high-altitude sheep; A2, the alternative allele; Significant SNPs ($P < 0.001$) are colored "red".

Talbe S12. Predicted Transcription Factor Binding Sites (TFBs) for the upstream mutation in *FGF-7*

**TFBIND prediction created binding sites**	**TFBIND prediction removed binding sites**
CEBPB (CCAAT enhancer-binding protein)	TAX/CREB
OCT (octamer-binding protein)	XBP1 (X-box binding protein 1)
YY1 (Yin Yang-1)	ARNT (AhR nuclear translocator)
IRF1 (Interferon regulatory factor)	CREBP1 (Activating transcription factor-2)
E2_Q6	CREB (cAMP response element binding element site)
	PBX1
	XFD1 (*Xenopus Fork* head Domain)
	HFH1 (*Fork* head homolog)
